# Bi_2_CoO_2_F_4_—A Polar, Ferrimagnetic Aurivillius Oxide-Fluoride

**DOI:** 10.1021/acs.chemmater.2c02745

**Published:** 2022-10-17

**Authors:** Euan A. S. Scott, Eleni Mitoudi Vagourdi, Mats Johnsson, Vanessa Cascos, Filbin John, Dave Pickup, Alan V. Chadwick, Hania Djani, Eric Bousquet, Weiguo Zhang, P. Shiv Halasyamani, Emma E. McCabe

**Affiliations:** † School of Physical Sciences, University of Kent, Kent, Canterbury CT2 7NH, U.K.; ‡ Department of Materials and Environmental Chemistry, Stockholm University, SE-106 91 Stockholm, Sweden; § Centre de Développement des Technologies Avancées, cité 20 aout 1956, Baba Hassan, Alger 16081, Algeria; ∥ Theoretical Materials Physics, Q-MAT, CESAM, Université de Liège, Allée 6 août, 17, B-4000, Sart Tilman, Liège 4000, Belgium; ⊥ Department of Chemistry, University of Houston, 112 Fleming Building, Houston, Texas 77204, United States; # Department of Physics, Durham University, South Road, Durham DH1 3LE, U.K.

## Abstract

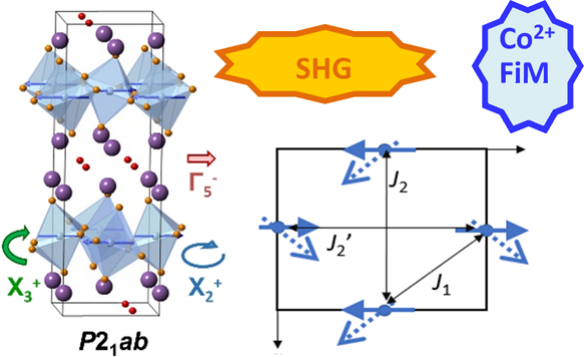

Aurivillius oxides have been a research focus due to their ferroelectric properties, but by replacing oxide ions by fluoride, divalent magnetic cations can be introduced, giving Bi_2_*M*O_2_F_4_ (*M* = Fe, Co, and Ni). Our combined experimental and computational study on Bi_2_CoO_2_F_4_ indicates a low-temperature polar structure of *P*2_1_*ab* symmetry (analogous to ferroelectric Bi_2_WO_6_) and a ferrimagnetic ground state. These results highlight the potential of Aurivillius oxide-fluorides for multiferroic properties. Our research has also revealed some challenges associated with the reduced tendency for polar displacements in the more ionic fluoride-based systems.

## Introduction

**Figure 1:**
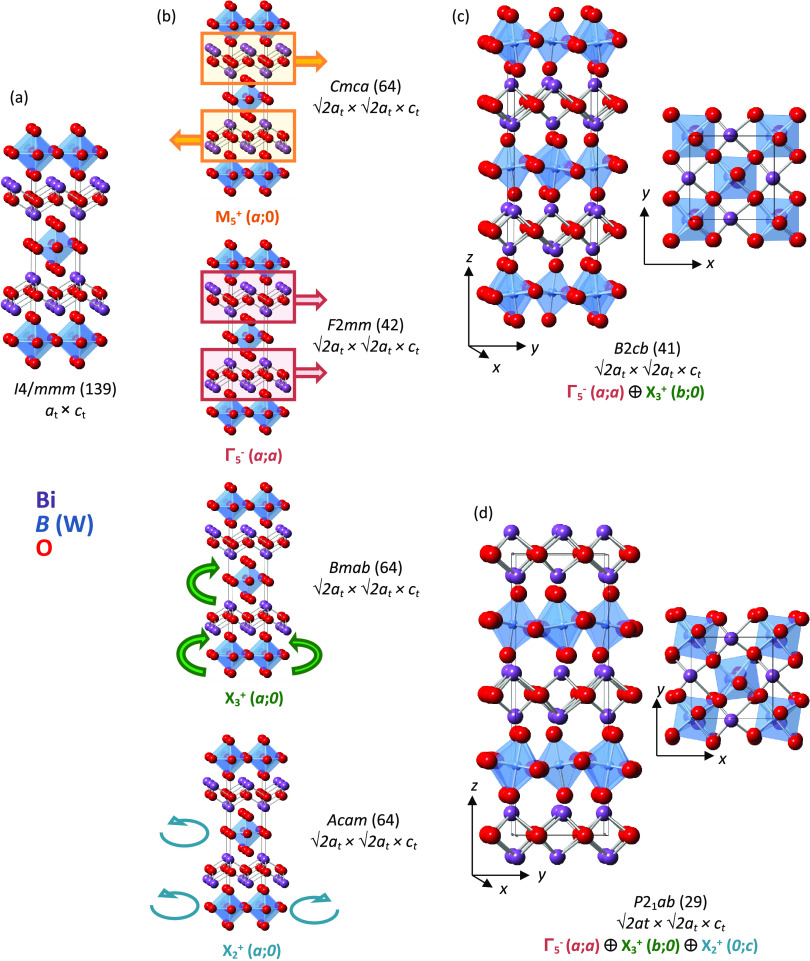
Illustration of (a) an ideal *I*4/*mmm* structure for an *n* = 1 Aurivillius oxide and (b) schematic showing key structural distortions (order parameter directions for the irreps are given in parentheses) for this structure (and resulting space group symmetries); panel (c) shows the intermediate *B*2*cb* structure of Bi_2_WO_6_ and panel (d) shows the low-temperature *P*2_1_*ab* model of Bi_2_WO_6_. Bi, B/W, and O ions are shown in purple, blue, and red, respectively.

Designing and preparing new multiferroic and magnetoelectric phases (with both polar and magnetic order) is an ongoing challenge in materials chemistry and physics.^1,2^ The Aurivillius oxides are well-known ferroelectrics,^3,4^ but it has proved challenging to introduce high concentrations of magnetic ions into these phases.^5−9^ Aurivillius materials, of general formula Bi_2_*A*_*n*–1_*B_n_*O_3*n*+3_ (with *A* typically group 2 or lanthanide or Bi, Pb cations; *B* typically d^0^ transition metal ions in high oxidation states), adopt layered perovskite-related structures composed of fluorite-like [Bi_2_O_2_]^2+^ layers separating perovskite-like blocks *n* layers thick (Figure 1). The ideal aristotype structure is tetragonal, but Aurivillius phases often adopt lower-symmetry distorted structures, which allow in-plane polarizations and rotations of *B*O_6_ octahedra.^7,10,11^ These distortions occur in part to optimize cation coordination environments and to relieve strain in stacking the slightly wider perovskite-like blocks with the more narrow [Bi_2_O_2_]^2+^ layers.^12^

These distortions can be discussed in the context of the *n* = 1 Aurivillius oxide Bi_2_WO_6_. Its aristotype high-symmetry structure would be of *I*4/*mmm* symmetry (*a*_t_ ≈ 3.8 Å, *c*_t_ ≈ 16.4 Å, where the subscript t denotes this ideal *I*4/*mmm* structure), although this ideal structure is never observed in practice—Bi_2_WO_6_ undergoes a reconstructive phase transition on warming above 950 °C.^13,14^ At intermediate temperatures, Bi_2_WO_6_ adopts a polar structure of *B*2*cb* symmetry, which allows rotations of WO_6_ octahedra about an in-plane axis (about [110]_t_), as well as polar displacements along this same axis (Figure 1).^13^ These distortions of odd-layer Aurivillius and Ruddlesden–Popper phases have been tabulated^15,16^ and can also be explored using web-based ISODISTORT software,^17^ and key distortions are illustrated in Figure 1 (see also the Supporting Information). The distortions can be described using irrep notation (where the letter denotes the *k*-point of the distortion, and the superscript indicates whether or not inversion symmetry is preserved), and the rotations of *B*O_6_ octahedra can also be described using Glazer notation.^18^ The intermediate *B*2*cb* structure of Bi_2_WO_6_ allows both X_3_^+^ (*a*^–^*a*^–^*c*^0^) rotations of WO_6_ octahedra and Γ_5_^–^ in-plane polar displacements.^13^ On cooling below 670 °C, a second WO_6_ rotation (X_2_^+^, *a*^0^*a*^0^*c*) occurs, lowering the symmetry further to *P*2_1_*ab*.^13,19^

The more limited compositional flexibility of the Aurivillius phases (including the difficulties associated with introducing magnetic ions) compared with perovskite and Ruddlesden–Popper systems may relate to steric factors related to stacking strain^12^ but could also arise from the polar displacements of the d^0^*B*-site cations in Aurivillius materials,^20^ which would be less favorable for magnetic d^*n*^ transition metal ions. Replacing some of the oxide ions by fluoride ions in *n* = 1 Aurivillius materials lowers the *B*-cation oxidation state required for charge balance^21−23^ and may also change the relative significance of polar displacements and rotations of octahedra.^24^ This has allowed magnetic *n* = 1 Aurivillius oxide-fluorides Bi_2_*B*O_2_F_4_ (*B* = Fe, Co, and Ni) to be prepared.^25−27^ The report of long-range magnetic order in Bi_2_CoO_2_F_4_ (with the possibility of a ferromagnetic component)^25^ motivated us to investigate it further. Mitoudi Vagourdi et al.^25^ were able to use X-ray diffraction to confirm its *n* = 1 Aurivillius structure, but X-rays are relatively insensitive to the positions of the light O/F anions, making it difficult to investigate possible structural distortions further. We report here the structural characterization of Bi_2_CoO_2_F_4_ using neutron powder diffraction (NPD) and second-harmonic generation (SHG) experiments, complemented by density functional theory (DFT) calculations. These methods reveal a distorted structure analogous to those adopted by ferroelectric Bi_2_WO_6_. Low-temperature NPD data allow us to determine the long-range magnetic structure of Bi_2_CoO_2_F_4_ and explain the field dependence reported by Mitoudi Vagourdi et al.^25^

## Methods

Pink polycrystalline samples of Bi_2_CoO_2_F_4_ were prepared hydrothermally, as described in ref (25). Variable temperature X-ray powder diffraction (XRPD) data were collected using a PANalytical Empyrean diffractometer with Cu K_α1_ radiation (with a Ge monochromator), an X’Celerator detector, and an Oxford Cryosystems PheniX cryostat. 20 min scans were collected at 20 K intervals on warming from 12 to 300 K, with dwell times of 10 min for temperature equilibration before each scan.

NPD data were collected at the ISIS Neutron and Muon Source. One batch (0.84 g) was used to collect medium-resolution NPD data on the GEM diffractometer; the sample was placed in a cylindrical vanadium sample can (diameter of 6 mm) to a height of ∼1 mm, and data were collected at 50 K and at 5 K. High-resolution NPD data were also collected on a second batch of sample (0.35 g) on the HRPD diffractometer; the sample was placed in a thin-walled vanadium can to a height of 1.9 cm, and data were collected at 5 K. Powder diffraction data were analyzed using Topas Academic software^28,29^ and ISODISTORT^30^ was used to explore possible structural distortions and magnetic ordering arrangements. Jana2006 software^31^ was used for incommensurate refinements.

X-ray absorption near-edge structure data was obtained on the Bi_2_CoO_2_F_4_ material using the B18-CORE XAS instrument at Diamond Light Source at the Harwell Science and Innovation Campus in Oxfordshire. XANES measurements of the Co K-edge were performed, and data was analyzed qualitatively to look at the oxidation state of the Co metal. Calibration of the energy scale was ensured by simultaneously collecting XANES data from a Co foil using a third ionization chamber.

Bi_2_CoO_2_F_4_ was tested for an SHG signal using the experimental setup described in ref (32); Bi_2_CoO_2_F_4_ powder was placed in a fused silica tube (with an outer diameter of 4 mm). Relevant comparisons with a known SHG material, α-SiO_2_, were made under the same conditions. A 1064 nm pulsed Nd:YAG laser (Quantel Laser, Ultra 50) generated the fundamental light, and the SHG intensity was recorded at room temperature using an oscilloscope (Tektronix, TDS3032).

Calculations were performed within DFT^33^ using the ABINIT package.^34,35^ The projected augmented waves (PAW) approach^36^ was used to represent the valence and core electrons. The exchange correlation energy functional was evaluated within both the local density approximation (LDA) and the generalized gradient approximation (GGA).^37^ The atomic data set was taken from the JTH table^38^ where 15 valence electrons were used for Bi (5d^10^ 6s^2^ 6p^3^), 17 for Co (3s^2^ 3p^6^ 3d^7^ 4s^2^), 6 for O (2s^2^ 2p^4^), and 7 for F (2s^2^ 2p^5^). The wave functions were expanded up to a maximum kinetic energy cutoff of 22 hartrees. Integrals over the Brillouin zone were approximated by sums on a 6 × 6 × 2 Monkhorst–Pack mesh of special *k*-points. Spin-polarized structural optimizations were performed in LDA and GGA until the absolute values of the forces on atoms were converged to less than 10^–5^ Ha/bohr. The 3d electrons were corrected through the DFT + U approximation^39,40^ where different values of on-site Coulomb interaction *U* and site exchange interaction *J* were used to check the energy differences between spin configurations. Phonons were calculated using density functional perturbation theory within GGA-JTH.^41,42^

## Results

### XANES

**Figure 2:**
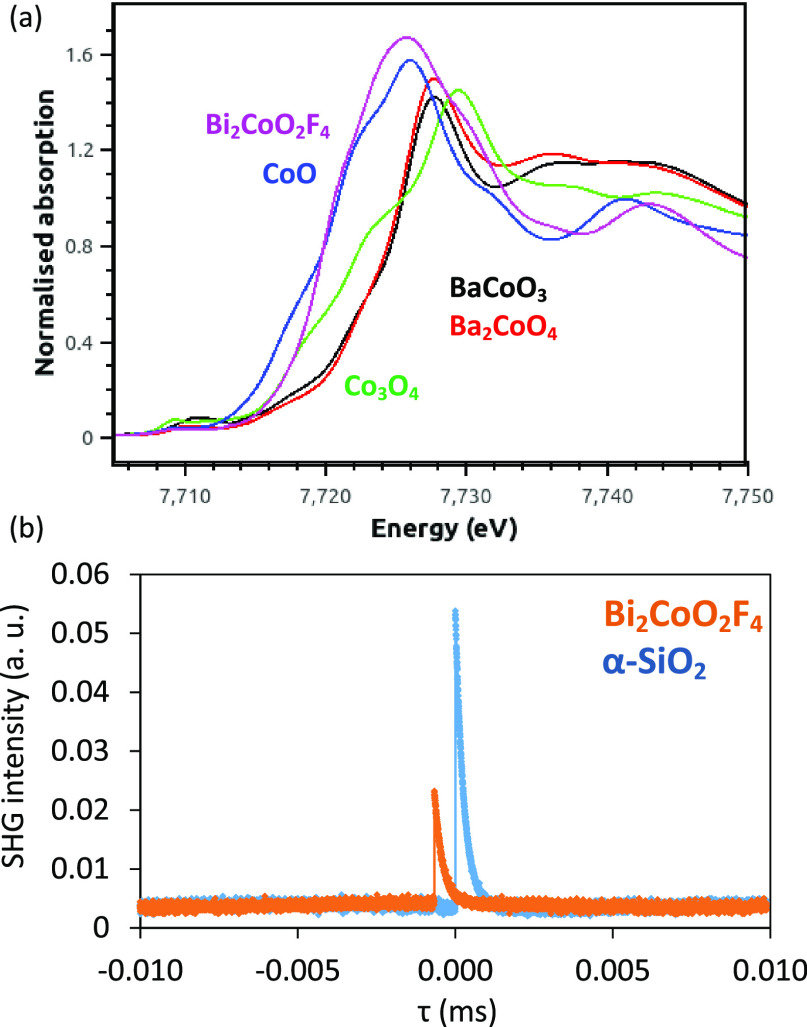
Panel (a) shows the X-ray absorption near-edge spectra (XANES) of Bi_2_CoO_2_F_4_ (pink), CoO (blue), Co_3_O_4_ (green), BaCoO_3_ and Ba_2_CoO_4_ (black and red) showing a cobalt oxidation state close to +2 in Bi_2_CoO_2_F_4_; panel (b) shows the SHG signal for Bi_2_CoO_2_F_4_ (orange) and compared with α-SiO_2_ (blue).

XANES is a useful tool for determining oxidation states of transition metals by comparison of the absorption edge position with that from standards containing the transition metal in known oxidation states. The energy of the absorption edge increases with the oxidation of state of the transition metal since more energy is required to remove electrons from ions with higher charge. In this case, CoO, Co_3_O_4_, Ba_2_CoO_4_, and BaCoO_3_ were chosen as standards with Co oxidation states of +2, +2.67, +4, and +4, respectively. Figure 2 shows the absorption edges of these reference materials and the Bi_2_CoO_2_F_4_ sample. As expected, the energy of the absorption edges of the standards increases with oxidation state. The energy of the absorption edge of the Bi_2_CoO_2_F_4_ sample, measured half way up the edge, is the same as that from CoO, indicating that the Co oxidation state in Bi_2_CoO_2_F_4_ is close to +2. We note that the Co site in Bi_2_CoO_2_F_4_ is thought to be coordinated by the more electronegative F^–^ anions rather than O^2–^ ions (see below and Mitoudi Vagourdi et al.^25^), which may shift the Co K-edge position to slightly higher energies.^43^ The observed edge position could therefore indicate a slightly lower cobalt oxidation state in our sample, but further work involving comparison with stoichiometric cobalt fluoride references is needed to confirm this.

### Second Harmonic Generation

SHG measurements using 1064 nm radiation gave a signal for Bi_2_CoO_2_F_4_ (about 0.4 times that of α-SiO_2_) (Figure 2); no laser damage was observed for the sample. This measurement indicates that Bi_2_CoO_2_F_4_ must adopt an acentric structure at room temperature.

### Preliminary Characterization Using XRPD Data

XRPD data were collected between 20 and 300 K to monitor unit cell parameters as a function of temperature. The data were fitted reasonably well by orthorhombic models considered for analysis of NPD data (see below), and the unit cell volume increased smoothly on warming (see the Supporting Information). There is a change in slope in the unit cell *c* parameter below 190 K (see the SI). This may indicate a structural change (e.g., a tilt transition) below this temperature as suggested from phonon analysis from Raman data,^25^ but further structural analysis as a function of temperature is needed to confirm this.

### Crystal Structure Determination from NPD Data

The main reflections observed in 50 K NPD data are consistent with an *n* = 1 Aurivillius phase, but additional reflections were also observed that cannot be indexed by the high-symmetry *I*4/*mmm* (*a*_t_ ≈ 3.8 Å, *c*_t_ ≈ 16.3 Å) cell. These additional reflections are consistent with a larger √2*a*_t_ × √2*a*_t_ × *c*_t_ unit cell. These superstructure reflections also rule out the non-centrosymmetric disordered model of *I*4̅ symmetry (*a* ≈ 3.8 Å; *c* ≈ 16.3 Å) reported by Mitoudi Vagourdi et al. from X-ray diffraction work.^25^ The observation of superstructure reflections from neutron scattering data (with relatively stronger sensitivity to the positions of the light anions)^44^ and not from careful X-ray studies (in which the scattering is dominated by the electron-rich Bi^3+^ sites) suggested that structural distortions involving displacements of the anion sites (such as rotations of the Co*X*_6_ octahedra) should be considered. (Given the similar neutron scattering lengths of oxygen and fluorine (5.803(4) and 5.65(1) fm, respectively),^44^ no attempt was made at this stage to determine the anion distribution over the three sites, and all were modeled as fully occupied by oxide ions.) ISODISTORT was used to explore possible distortions that might give rise to such a supercell. Mode inclusion analysis^45−47^ (see the Supporting Information) suggested that the lower-symmetry structure results from rotation of Co*X*_6_ octahedra about an in-plane axis (described by irrep X_3_^+^; see Figure 1 and the Supporting Information), giving a model of *Bmab* symmetry (*ac̅b* setting of space group 54, *Cmca*).

The SHG activity observed (Figure 2) indicates that Bi_2_CoO_2_F_4_ must adopt a non-centrosymmetric crystal structure, and so non-centrosymmetric, polar models (including those of *B*2*cb* and *P*2_1_*ab* symmetry, analogous to Bi_2_WO_6_)^13^ were considered. This was consistent with mode inclusion analysis, which suggested a further improvement in fit if in-plane polar displacements (described by the Γ_5_^–^ irrep), or rotation of Co*X*_6_ octahedra about the long axis ([001]_t_), described by the X_2_^+^ irrep, were allowed (see the Supporting Information).

The model of *B*2*cb* symmetry (*cab* setting of space group 41, *Aba*2) allows displacements along the polar *a* axis ([110]_t_, where the subscript t denotes the high-symmetry *I*4/*mmm* unit cell), as well as rotation of Co*X*_6_ octahedra about this axis (described by the X_3_^+^ irrep). Lowering the symmetry further to *P*2_1_*ab* (*cab* setting of space group 29, *Pca*2_1_) allows an additional rotation of Co*X*_6_ octahedra about the long axis ([001]_t_) described by the X_2_^+^ irrep.

**Figure 3:**
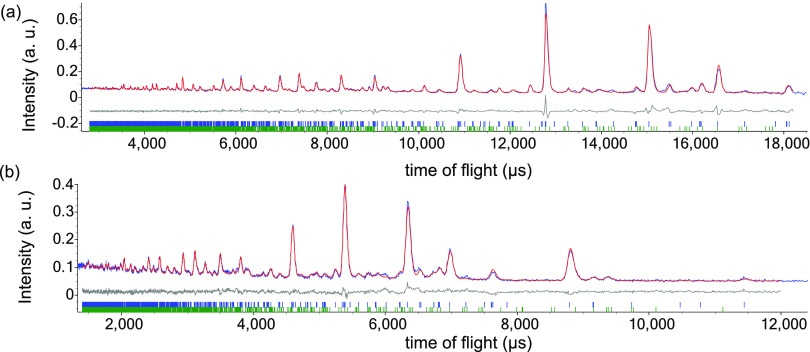
Details from Rietveld refinement using 50 K NPD data collected for Bi_2_CoO_2_F_4_ using the *P*2_1_*ab* model with anion vacancies. The refinement was carried out using NPD data from the 91° (panel a) and 35° (panel b) banks with upper ticks (blue) and lower ticks (green) showing peak positions for Bi_2_CoO_2_F_4_ (86(1)% by mass) and for Bi_7_F_11_O_5_ (14(1)% by mass), respectively; R_wp_ = 6.32%, R_p_ = 4.64%, and χ^2^ = 2.90%.

Both *B*2*cb* and *P*2_1_*ab* models give good fits to the data at 50 K (R_wp_ of 6.64% (61 parameters) and 6.28% (79 parameters) for *B*2*cb* and *P*2_1_*ab* models, respectively) (see the Supporting Information) but surprisingly high atomic displacement parameters for the equatorial anion site(s). In Rietveld refinements, atomic displacement parameters are often correlated with site occupancies, and so this could indicate vacancies on this site or a problem with the model in terms of the position of the site. For both models, refinements were carried out using a single global atomic displacement parameter and allowing occupancies of anion and cobalt sites to refine, while the bismuth site occupancies were fixed at unity. These refinements suggested significant vacancies on equatorial anion sites (other site occupancies refined to unity with two esds and so were fixed as fully occupied), similar to reports for LaSrCoO_3.5–x_.^48^ (Site occupancies refined to values of 0.75(4), 0.76(4), and 0.81(1) for X(1), X(2), and X(4) sites, respectively.) Site occupancies were fixed at these values for subsequent refinements, and refining atomic displacement parameters for each site gave more physically reasonable values, and so it is likely that there are some anion vacancies in this sample of Bi_2_CoO_2_F_4_. Allowing the atomic displacement parameter to refine anisotropically for this site in the *B*2*cb* model did suggest some increased displacement (either static or dynamic) in the *ab* plane, which may indicate some further rotation of Co*X*_6_ octahedra (e.g., about the long axis). Refinement details for *P*2_1_*ab* refinements using 50 K data are shown in Figure 3 and Table 1 and below (Table 2 and Figures 5 and 6) for refinements using 5 K data.

**Table 1:** Details from Rietveld Refinement Using 50 K NPD Data Collected for Bi_2_CoO_2_F_4_ Using the *P*2_1_*ab* Model with Anion Vacancies^b^

atom	site	*x*	*y*	*z*	occupancy	*U*_iso_ × 100 (Å^3^)
Bi(1)	4*a*	0.004(6)	0.011(2)	0.0776(7)	1	1.7(2)
Bi(2)	4*a*	0.505(6)	0.009(2)	0.5771(7)	1	1.4(1)
Co	4*a*	0^a^	0.976(4)	0.747(1)	1	0.6(2)
O/F(1) (eq)	4*a*	0.208(7)	0.290(3)	0.7865(7)	0.75	1.5(2)
O/F(2) (eq)	4*a*	0.806(7)	0.199(4)	0.2700(9)	0.76	2.7(4)
O/F(3) (ap)	4*a*	0.027(7)	0.929(4)	0.874(1)	1	2.3(3)
O/F(4) (ap)	4*a*	0.517(7)	0.933(4)	0.373(1)	0.81	2.0(4)
O/F(5) (fl)	4*a*	0.255(7)	0.243(3)	0.994(6)	1	1.7(3)
O/F(6) (fl)	4*a*	0.749(7)	0.256(3)	0.4996(6)	1	1.4(2)

a Coordinate fixed to define the origin of the polar axis.

b The refinement was carried out using NPD data from the 91 and 35° banks and included Bi_7_F_11_O_5_ impurity (14(1)% by mass). The Bi_2_CoO_2_F_4_ main phase (86(1)% by mass) had unit cell parameters *a* = 5.4343(5) Å, *b* = 5.4339(6) Å, *c* = 16.350(1) Å and volume = 482.81(8) Å^3^; R_wp_ = 6.32%, R_p_ = 4.64%, and χ^2^ = 2.90%. (Abbreviations “ap”, “eq”, and “fl” refer to “apical”, “equatorial”, and “fluorite”, respectively.)

**Table 2:** Details from Rietveld Refinement Using 5 K NPD Data Collected for Bi_2_CoO_2_F_4_ Using the *P*2_1_*ab* Model (with Anion Vacancies) and mΓ_2_ Magnetic Model^b^

atom	site	*x*	*y*	*z*	occupancy	*U*_iso_ × 100 (Å^3^)
Bi(1)	4*a*	0.006(4)	0.010(2)	0.0780(5)	1	0.1(1)
Bi(2)	4*a*	0.508(5)	0.009(2)	0.5767(6)	1	0.5(1)
Co	4*a*	0^a^	0.980(4)	0.748(1)	1	0.2(2)
F(1)	4*a*	0.207(5)	0.290(3)	0.7847(7)	0.75	0.0(1)
F(2)	4*a*	0.805(6)	0.198(4)	0.2712(9)	0.76	1.9(4)
F(3)	4*a*	0.024(5)	0.928(3)	0.873(1)	1	1.3(3)
F(4)	4*a*	0.519(6)	0.933(4)	0.374(1)	0.81	0.9(4)
O(5)	4*a*	0.252(5)	0.241(2)	0.9941(6)	1	0.5(2)
O(6)	4*a*	0.743(5)	0.254(2)	0.4991(6)	1	0.1(1)

a Coordinate fixed to define the origin of the polar axis.

b The refinement was carried out using NPD data from the 91 and 35° banks and included Bi_7_F_11_O_5_ impurity (14% by mass). The Bi_2_CoO_2_F_4_ main phase (86% by mass) has unit cell parameters *a* = 5.4317(5) Å, *b* = 5.4313(5) Å, *c* = 16.348 (1) Å and volume = 482.30(7) Å^3^ with Co^2+^ moments of 2.5(1) μ_B_ (2.37(1) μ_B_ along *b* and 0.7(2) μ_B_ along *a*); R_wp_ = 5.83%, R_p_ = 3.94%, and χ^2^ = 14.2%.

Bond valence sum calculations were used as a simplistic way to determine the likely anion distribution using 50 K bond lengths. (Bond valence sum parameters calculated for this low-temperature model should not be interpreted quantitatively as the parameters are determined from room temperature structures,^49,50^ but can be instructive for comparing between different ordering models.) Calculating apparent valences for equatorial and apical sites gave valences close to 1 (assuming either F or O occupancy), while higher valences closer to 2 were calculated for anion sites in the fluorite-like layers (full details are given in the Supporting Information). These values suggest that anion sites in the perovskite layers might be favored by F^–^ anions giving CoF_6_ octahedra, while O^2–^ ions occupy sites in the fluorite-like [Bi_2_O_2_]^2+^ layers. Taking into account the anion vacancies noted above, this suggests an approximate composition of Bi_2_CoO_2_F_3.32_ compared with the target stoichiometry of Bi_2_CoO_2_F_4_.

### Magnetic Structure Determination from 5 K NPD Data

NPD data collected (on the GEM diffractometer) at 5 K were similar to those observed at 50 K, with some additional low-intensity peaks observed at long d-spacing (see the Supporting Information). Most of these peaks could be indexed by the same size unit cell as the nuclear structure, although a shoulder to the 102 peak (∼12,700 μs, 4.52 Å) was not indexed by this unit cell. Attempts to index this peak using larger or lower-symmetry unit cells were unsuccessful.

High-resolution NPD data were also collected at 5 K, and although the strong reflections were consistent with those observed in the GEM data, some additional reflections on either side of some strong reflections were also observed. Attempts were made to fit these satellite reflections, but the reflections were fairly broad and low intensity, and our attempts were not successful (stable refinements were not achieved). It is likely that they indicate an incommensurate modulation of the crystal structure, but their relatively broad nature may indicate a shorter correlation length of this modulation compared with the long-range average (commensurate) structure. Evidence for an incommensurate modulation in the crystal structure of another sample of Bi_2_CoO_2_F_4_ has also been observed in electron diffraction data,^51^ but the possibility of vacancies on the anion sublattice, and slight differences in the O:F ratio, means that this incommensurate modulation could be quite sample-dependent. Our subsequent analysis is based on the average commensurate structure as determined from our NPD data collected on the GEM diffractometer.

**Figure 4:**
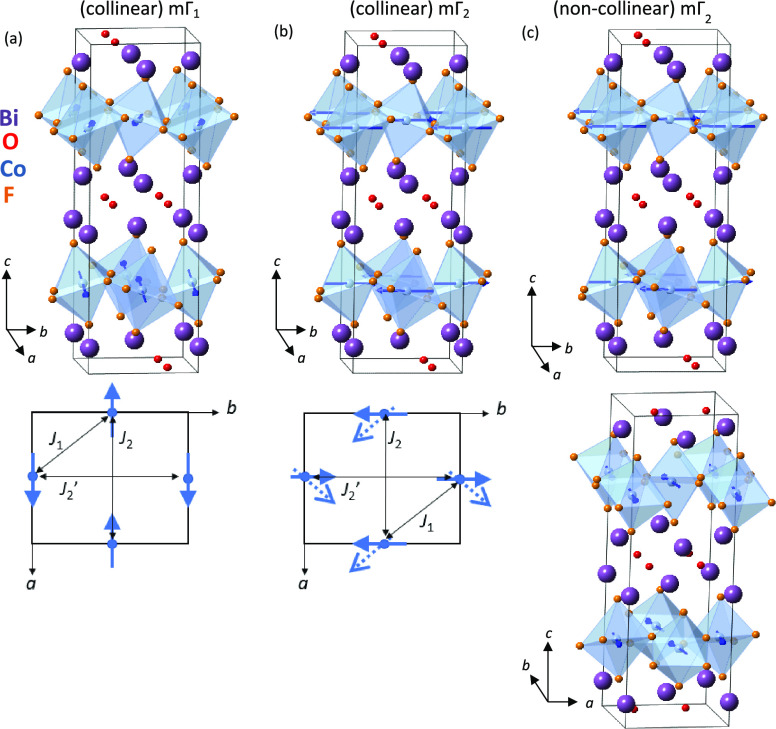
Schematic illustration of the *P*2_1_*ab* nuclear structure comparing (a) the collinear mΓ_1_ magnetic structure and (b) the collinear mΓ_2_ magnetic structure and (c) the final experimental *P*2_1_*ab* nuclear structure and the noncollinear mΓ_2_ magnetic structure from refinements using 5 K NPD data. CoF_6_ polyhedra are shown in pale blue, and Co^2+^ moments are shown by blue arrows; Co, Bi, F, and O sites are shown by blue, purple, orange, and red spheres, respectively. The bottom panels illustrate the approximately collinear arrangement of Co^2+^ moments (blue arrows) with the *ab* plane (with the noncollinear mΓ_2_ model with FM component shown by dashed arrows), with the orthorhombic distortion exaggerated. nn *J*_1_ and nnn *J*_2_ and *J*_2_’ interactions are also illustrated.

**Figure 5:**
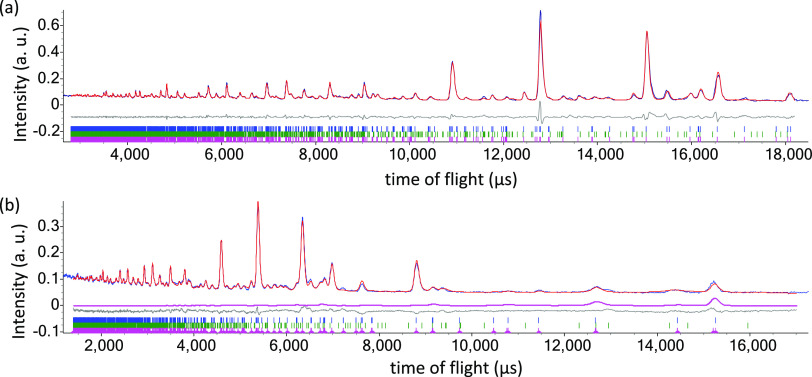
Details from Rietveld refinement using 5 K NPD data collected for Bi_2_CoO_2_F_4_ using the *P*2_1_*ab* model (with anion vacancies) and mΓ_2_ magnetic model. The refinement was carried out using NPD data from the 91° (panel a) and 35° (panel b) banks with upper ticks (blue) and middle ticks (green) showing peak positions for Bi_2_CoO_2_F_4_ (86% by mass) and for Bi_7_F_11_O_5_ (14% by mass), respectively; bottom ticks (pink) show positions of magnetic peaks, and the magnetic scattering is highlighted in pink. R_wp_ = 5.83%, R_p_ = 3.94%, and χ^2^ = 14.2%.

ISODISTORT was used to explore possible magnetic structures to fit the 5 K (GEM) NPD data, assuming a *P*2_1_*ab* nuclear structure. Mode inclusion analysis (see the Supporting Information) suggested that models with an antiferromagnetic arrangement of moments in-plane gave good fits to the data. Two almost collinear models, mΓ_1_ and mΓ_2_ (see Figure 4a,b), give comparable fits to the data, and it is difficult to differentiate between these models from NPD data. The mΓ_1_ model has Co^2+^ moments along the polar *a* axis, which is the axis about which CoF_6_ octahedra rotate, similar to the intermediate-temperature magnetic model reported for La_2_CoO_4_ (with nuclear symmetry *Bmab*).^52^ The mΓ_2_ model is similar but with Co^2+^ moments predominantly along the in-plane axis perpendicular to this (see Figure 4a,b). Given the tiny orthorhombic distortion observed, it is not surprising that these models give similar fits to our NPD data, and it is likely that these models are of similar energies. Interestingly, the mΓ_2_ model allows an FM in-plane component (perhaps consistent with the low-field magnetic behavior evidenced by magnetization vs field measurements reported by Mitoudi Vagourdi et al.).^25^ Further diffraction studies on single crystals (e.g., susceptibility measurements on single crystals or polarized neutron diffraction experiments) might allow these two models to be distinguished. Allowing this FM in-plane component to refine for the mΓ_2_ model gave a stable refinement (although only small improvement in fit; R_wp_ decreased from 5.826 to 5.825% for this additional parameter), and a small nonzero FM component along [100] was obtained (0.7(2) μ_B_), alongside the AFM component along [010] (2.37(7) μ_B_), giving a total moment per Co^2+^ site of 2.5(1) μ_B_ (further refinement details are given in the Supporting Information). Refinement details are given in Table 2 and profiles in Figure 5, with the final nuclear and magnetic structure illustrated in Figure 4c.

### Density Functional Theory Calculations

Initial phonon calculations on the parent *I*4/*mmm* model were used to determine key structural instabilities (to identify structural models for further spin-polarized calculations). Six unstable modes (which are all doubly degenerate) were identified: X_2_^+^ and X_3_^+^ rotations, Γ_5_^–^ and M_5_^+^ polar and antipolar in-plane displacements (Figure 1), as well as another rotation mode X_4_^+^ (*a*^–^*a*^–^*c*^0^/–(*a*^–^*a*^–^*c*^0^)) and X_3_^–^ in-plane antipolar displacements (see the Supporting Information). It is striking that the “rigid-layer” modes Γ_5_^–^ and M_5_^+^, which describe in-plane displacements of [Bi_2_O_2_]^2+^ layers relative to the perovskite-like blocks (along [110]_t_), are harder than often observed in perovskite-related Aurivillius phases.

Spin-polarized calculations were therefore carried out for the aristotype *I*4/*mmm* model and for lower-symmetry models that allow these distortions (and are common ground states for *n* = 1 Aurivillius phases):

*Pbca* (X_3_^+^ ⊕ X_2_^+^ ⊕ M_5_^+^)
*B*2*cb* (X_3_^+^ ⊕ Γ_5_^–^)
*P*2_1_*ab* (X_3_^+^ ⊕ X_2_^+^ ⊕ Γ_5_^–^)

**Figure 6:**
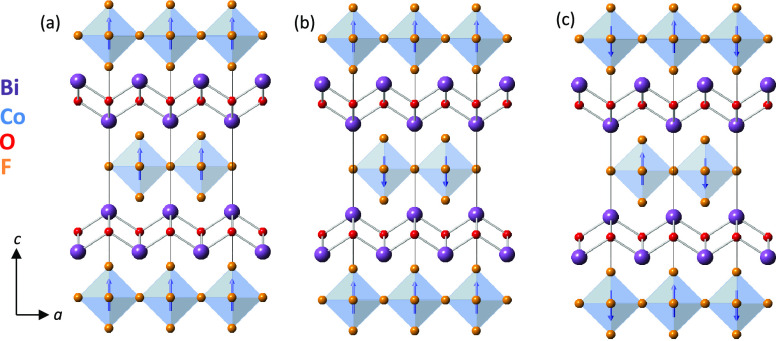
Schematic showing the three magnetic structures considered in spin-polarized DFT calculations: (a) FM, (b) AFM1 (with ferromagnetic intralayer interactions and antiferromagnetic interlayer interactions), and (c) AFM(2) with intralayer and interlayer coupling both antiferromagnetic. CoF_6_ polyhedra are shown in pale blue, and Co^2+^ moments are shown by blue arrows; Co, Bi, F, and O sites are shown by blue, purple, orange, and red spheres, respectively. (Anions are shown as small spheres for clarity.)

Three arrangements of Co^2+^ magnetic moments (Figure 6) were considered for each of the three nuclear structures: FM with all moments ferromagnetically aligned; AFM1 with ferromagnetic alignment within CoF_4_ layers and antiferromagnetic coupling between layers; and AFM2 with antiferromagnetic CoF_4_ layers. Preliminary calculations using LDA-JTH were inconclusive, being very sensitive to geometry optimization and giving results inconsistent with the experiment (see the Supporting Information).

Calculations using GGA-JTH gave the FM ground state for the *I*4/*mmm* nuclear structure, but AFM2 ground states (with AFM1 next lowest in energy) for all the orthorhombic nuclear structures (for *U* = 4 and 6 eV; *J* = 0.2, 0.4, and 0.6 eV), consistent with experimental results. Having established AFM2 as the ground state magnetic structure, it is interesting to compare the three nuclear models with the aristotype *I*4/*mmm* model (with AFM2 spin order) (Table 3).

**Table 3:** Relative Energies ΔE (meV per Formula Unit) and Mode Contributions (Calculated by AMPLIMODES)^53,54^ to the AFM2 Ground State Models for Bi_2_CoO_2_F_4_ Relative to the *I*4/*mmm* (AFM2) Model, for *U* = 4 eV and *J* = 0.6 eV

nuclear structure	mode contributions (Å)	ΔE (meV/f.u.)
*I*4/*mmm*		0
*Pbca*	1.86 X_3_+ ⊕ 1.14 X_2_+ ⊕ 0.41 M_5_+	–480
*B*2*cb*	1.37 X_3_+ ⊕ 0.011 Γ_5_–	–871
*P*2_1_*ab*	2.02 X_3_+ ⊕ 1.2 X_2_+ ⊕ 0.011 Γ_5_– ⊕ 0.59 M_5_+	–874

These results from DFT calculations are largely consistent with the experimental results discussed above, in terms of both the nuclear and magnetic structures.

## Discussion

Analysis of NPD data collected at 50 K suggests that “Bi_2_CoO_2_F_4_” may be slightly nonstoichiometric with anion vacancies. If these vacancies involve only F^–^ ions, the refined occupancies would suggest a composition closer to Bi_2_CoO_2_F_3.32_. This is reminiscent of reports on La_2_CoO_3.86_ (prepared by the floating zone method under controlled atmosphere),^55^ suggesting that cobalt ions may adopt an oxidation state <2. This contrasts with a recent report on “Bi_2_*M*O_2_F_4_” (*M* = Fe and Ni) analogues in which the stability of the +2 oxidation state for nickel gives stoichiometric Bi_2_NiO_2_F_4_, while the iron analogue is Bi-deficient with Fe^+2/+3^ cations.^27^ Given that the XANES data suggest a cobalt oxidation state close to +2 (Figure 2), the possibility of some excess oxygen and a small degree of O–F anti-site disorder (O^2–^ ↔ 2F^–^, giving compositions Bi_2_CoO_2+*x*/2_F_4–*x*_) seems likely (i.e., for the sample described in Table 1, the site occupancies would suggest *x* ≈ 1.38, Bi_2_CoO_2.69_F_2.62_). This would result in partial occupancy of some nominally “F^–^” sites by vacancies and O^2–^ ions, consistent with site occupancies from NPD analysis (Table 1). The anion vacancies are largely concentrated on the equatorial sites, consistent with LaSrCoO_3.5–*x*_ in which equatorial oxide vacancies occur to maintain the expected Co^2+^ oxidation state.^48^ This illustrates the difficulty in preparing pure, stoichiometric Aurivillius oxyfluorides containing d*^n^B*-site cations. It is possible that the precise composition varies from sample to sample (and between batches) and may depend on the synthetic route.

The 50 K (above *T*_N_) crystal structure of Bi_2_CoO_2_F_4_ is orthorhombic, but it is striking that the unit cell is metrically very close to tetragonal (*a* = 5.4343(5) Å, *b* = 5.4339(6) Å), as reported for other *n* = 1 Aurivillius oxyfluorides (including Bi_2_NbO_5_F, Bi_1.8_FeO_2_F_4_, and Bi_2_NiO_2_F_4_).^22,23,27^ The orthorhombic crystal structure of Bi_2_CoO_2_F_4_ of *P*2_1_*ab* symmetry can be described in terms of rotations of CoF_6_ octahedra about the *a* axis (X_3_^+^ irrep; *a*^–^*a*^–^*c*^0^ in Glazer notation relative to the parent structure of *I*4/*mmm* symmetry) and about the long axis (X_2_^+^ irrep; *a*^0^*a*^0^*c* in Glazer notation), as well as small polar displacements of cations relative to anions along the polar *a* axis, as mapped out in the Supporting Information.^17^ Our analysis of diffraction data and the observed SHG activity indicate that Bi_2_CoO_2_F_4_ adopts a crystal structure allowing both polar Γ_5_^–^ displacements as well as rotation of CoF_6_ about an in-plane axis (X_3_^+^ rotations).

Results from DFT calculations (Table 3 and the Supporting Information) are consistent with the experimental models described above, with lower-symmetry models calculated for all magnetic models dominated by X_3_^+^ (*a*^–^*a*^–^*c*^0^) rotations of CoF_6_ octahedra, with some further stabilization (and smaller contributions) from polar displacements (Γ_5_^–^) and X_2_^+^ (*a*^0^*a*^0^*c^n^*) rotations.

If the only long-range ordered distortions are X_3_^+^ rotations and polar Γ_5_^–^ displacements, then the model of *B*2*cb* symmetry would seem most appropriate to describe the average long-range structure. It is difficult to confirm the *P*2_1_*ab* structure (with long-range order of X_2_^+^ rotations) over a disordered model of *B*2*cb* symmetry from our powder diffraction data, and a larger sample of higher purity would be needed for high-resolution NPD data or complementary electron diffraction studies. DFT results (Supporting Information) indicate that the X_2_^+^ mode is significantly softer than the polar Γ_5_^–^ instability, and so the *P*2_1_*ab* (X_3_^+^ ⊕ X_2_^+^ ⊕ Γ_5_^–^) model seems the best description of the ground state. The difficulty in distinguishing between the disordered *B*2*cb* and ordered *P*2_1_*ab* models experimentally is perhaps explained by their similar energies calculated by DFT (Table 3 and Supporting Information). It is possible that the second rotation mode, X_2_^+^, condenses in on cooling, consistent with the disorder–order transition on cooling noted from Raman spectroscopy.^25^

The Ruddlesden–Popper phase La_2_CoO_4_ (and related analogues) adopts a closely related structure of *Bmab* symmetry (for 135 K < *T* < 408 K) with rotation of CoO_6_ octahedra about the in-plane axis (X_3_^+^ rotations) but without further rotations or polar displacements.^52,56,57^ On further cooling, La_2_CoO_4_ undergoes a structural phase transition to tetragonal *P*4_2_/*ncm* symmetry, in which the CoO_6_ rotation axis (in-plane) rotates by 90° in successive layers.^52,58^ Models of *P*4_2_/*ncm* symmetry were also considered for Bi_2_CoO_2_F_4_ but gave fairly poor fits to the data (there was no clear change in peak widths from data at 50 K and at 5 K that might indicate a similar orthorhombic–tetragonal distortion for Bi_2_CoO_2_F_4_). La_2_CoO_4_ contrasts with the Aurivillius oxide Bi_2_WO_6_ that adopts a polar ground state of *P*2_1_*ab* symmetry (allowing X_3_^+^ and X_2_^+^ rotations as well as Γ_5_^–^ polar displacements).

The comparison between polar Bi_2_WO_6_ and Bi_2_CoO_2_F_4_ with nonpolar Ruddlesden–Popper La_2_CoO_4_ perhaps highlights the role of the fluorite-like [Bi_2_O_2_]^2+^ layers in the *n* = 1 Aurivillius phases that contain 6s^2^ Bi^3+^ cations, known to favor lower-symmetry coordination environments.^59^ However, while the Bi^3+^ cations may help stabilize these further distortions (X_2_^+^ tilts and polar displacements), the dominant contribution to the polarization is from the perovskite-like layers (apical F^–^ displacements and, to a lesser extent, Co^2+^ and equatorial F^–^ displacements), consistent with theory work on analogous Bi_2_WO_6_.^20^ Polar displacements of d^0^ cations are often ascribed to the pseudo-Jahn–Teller effect,^60−62^ and this electronic driving force is lessened for d^*n*^ cations such as Co^2+^ here. Morita et al. explored the change in polarization with F-content in the series Bi_2_(W,*B*)(O,F)_6_ (*B* = Ta, Nb, and Ti) and noted that the polarization decreased noticeably with increasing F^–^ content.^24^ This is likely explained by reduced cation–anion hybridization for more ionic fluoride-based systems,^63^ increasing the importance of geometric drivers for distortions over electronic (pseudo-Jahn–Teller effect) factors.^64^ This is consistent with polar Γ_5_^–^ distortions being the softest instability for Bi_2_WO_6_ (and making similar contributions as X_3_^+^ and X_2_^+^ rotations to the ground state),^65^ while the X_3_^+^ rotations are dominant for Bi_2_CoO_2_F_4_ and Γ_5_^–^ distortions make a much diminished contribution (Table 3 and Supporting Information). The reduced polarization as oxide ions are replaced by fluoride might also be a factor in the smaller orthorhombic distortion of the oxide-fluorides.^22,23,27^

When analyzing the 5 K NPD data, we have tentatively described possible magnetic structures, assuming the *P*2_1_*ab* nuclear structure (on the assumption that the second tilt mode condenses in on cooling), but similar magnetic models can be derived for the *B*2*cb* nuclear structure (see the Supporting Information). Our analysis of the 5 K NPD data suggests that Co^2+^ moments are close to collinear (in zero applied magnetic field) with antiferromagnetic nearest-neighbor interactions, consistent with the negative Weiss temperature (θ = −142(2) K) determined by Mitoudi Vagourdi et al. (for *T* > 150 K in 7 T applied field).^25^ The magnetic peaks are slightly broadened (particularly those with large *l* index) compared with the nuclear Bragg peaks, and this was fitted using a model to describe antiphase boundaries^47,66^ in the magnetic structure perpendicular to the *c* axis, with a magnetic correlation length ξ_c_ ≈ 60(10) Å at 5 K. This suggests that the in-plane magnetic exchange interactions are noticeably stronger than the interlayer exchange interactions. This is unsurprising given the layered crystal structure and is consistent with the 2D-like character reported for La_2_CoO_4_.^52,67^ The size of the ordered Co^2+^ moment (∼2.5 μ_B_) is comparable to that reported for La_2_CoO_4_ (2.9 μ_B_)^52^ and close to the spin-only value often observed for high-spin Co^2+^ d^7^ ions (in elongated D_4h_ coordination environments).^68^ The magnetic models determined here for Bi_2_CoO_2_F_4_ are close to the collinear AFM models described for the *Bmab* phases of La_2_CoO_4_ and La_2_NiO_4_,^52^ with moments (close to) pointing toward an equatorial edge of the CoF_6_ octahedra. Similar magnetic structures are reported for LaSrFeO_4_^69,70^ and La_2_NiO_4_^52^ with Fe^3+^/Ni^2+^ spins in-plane and pointing toward an equatorial edge of the *B*O_6_ octahedra. We note that the intermediate (*Bmab*) phase reported for (stoichiometric) La_2_NiO_4_ has Ni^2+^ moments directed along the axis of the tilts of NiO_6_ octahedra,^52,71^ similar to the mΓ_1_ model described here for Bi_2_CoO_2_F_4_. The temperature below which 3D magnetic order emerges in Bi_2_CoO_2_F_4_ (∼75 K) is significantly lower than that for La_2_CoO_4_ (275 K), presumably reflecting the weakened interlayer interactions in Bi_2_CoO_2_F_4_ due to the larger separation of Co^2+^ layers by fluorite-like [Bi_2_O_2_]^2+^ layers (∼8.1 Å at 5 K in Bi_2_CoO_2_F_4_; ∼6.1 Å at 10 K in La_2_CoO_4_).^72^

Our magnetic structure refinements for Bi_2_CoO_2_F_4_ (see above and the Supporting Information) suggest that Co^2+^ moments are close to in-plane, consistent with the largely XY-like anisotropy reported for La_2_CoO_4_.^67^ Inelastic neutron scattering work has found that the magnetism in La_2_CoO_4_ is dominated by AFM nearest-neighbor (nn) interactions (*J*_1_ in Figure 4), while next-nearest-neighbor (nnn) interactions (*J*_2_, *J*_2_’) are much weaker. This is consistent with the mΓ_1_ and mΓ_2_ models for Bi_2_CoO_2_F_4_ that both allow AFM nn interactions (with the result that nnn pathways are FM and therefore frustrated).

There is a small Ising anisotropy reported for La_2_CoO_4_ that stabilizes the collinear magnetic model observed for the *Bmab* phase (135 < *T* < 275 K), but this in-plane anisotropy is sufficiently small that on further cooling, the Co^2+^ moments rotate by 90° in successive layers (accompanied by an orthorhombic–tetragonal phase transition). Similar magnetocrystalline anisotropy might be expected for Bi_2_CoO_2_F_4_, but the small orthorhombic distortion is likely to give little energy difference between the mΓ_1_ and mΓ_2_ models (with moments predominantly along [100] and [010] directions, respectively, of the *P*2_1_*ab* nuclear unit cell). Canting of the moments away from the collinear models may result from crystal field anisotropy of the Co^2+^ ions or from Dzyaloshinskii–Moriya (DM) exchange interactions, which would not be possible for the parent nuclear structure of *I*4/*mmm* symmetry (with inversion centers along the nn Co–F–Co superexchange pathways).^73,74^ The magnetic field dependence observed in susceptibility measurements for Bi_2_CoO_2_F_4_^25^ suggests that an applied magnetic field could also change the balance between these terms in the Hamiltonian. Measurements carried out in low magnetic fields show significant field dependence, consistent with a small ferromagnetic component (as might arise from canting of the moments, similar to that reported for Sr_2_IrO_4_^75^), consistent with the mΓ_2_ model. As the applied magnetic field increases, this FM component is reduced and Bi_2_CoO_2_F_4_ behaves more like a collinear AFM (consistent with the mΓ_1_ model).^25^ It seems that an increased applied magnetic field stabilizes the collinear AFM mΓ_1_ model for Bi_2_CoO_2_F_4_ rather than the (canted) ferrimagnetic mΓ_2_ model observed at low fields. Theory work to explore the relative energies of the exchange interactions and sources of anisotropy in Bi_2_CoO_2_F_4_, as well as characterization using single crystals, would give further insights into this.

## Conclusions

Our combined experimental and computational studies on Bi_2_CoO_2_F_4_ have given insight into its structural and magnetic properties. The ground state structure is best described by polar *P*2_1_*ab* symmetry, analogous to the Aurivillius oxide ferroelectric Bi_2_WO_6_.^13,19^ The magnetic ground state of Bi_2_CoO_2_F_4_ is ferrimagnetic, arising from a small canting of otherwise collinear AFM arrangement of moments. This combination of polarity and FM component highlights the potential of Aurivillius oxide-fluorides for multiferroics and magnetoelectrics, particularly given the magnetostrictive effects observed at *T*_N_ in capacitance data for Bi_2_FeO_2_F_4_.^27^ However, our work has also identified some challenges: while replacing O^2–^ by F^–^ ions allows more magnetic ions to be accommodated by the perovskite-like layers (and gives similar magnetic behavior to the Ruddlesden–Popper oxides such as La_2_CoO_4_),^52,67^ the increased ionic character of the fluorides compared with oxides tends to reduce the stability of polar distortions, reducing the polarization.^24^ It would be interesting to study the effect of concentrating F^–^ ions in the equatorial sites in the perovskite-like layers, leaving the apical sites fully occupied by O^2–^.^76^ This may give similar energies for the polar distortions involving [Bi_2_O_2_]^2+^ layers to those observed in Bi_2_WO_6_ (although the polarization contribution from the perovskite-like layers is likely to remain diminished). Future work to explore the optimum O:F ratio and ordering for designing multiferroic and magnetoelectric Aurivillius oxide-fluorides would be valuable.

## Supporting Information Available

Analysis of variable temperature XRPD data, symmetry analysis for *n* = 1 Aurivillius phases, additional refinements (Rietveld and Pawley) using different models for 50 K and 5 K NPD data, selected bond lengths (and bond valence sum analysis) and angles from Rietveld refinements using NPD data, details of the magnetic structure investigation, and details from DFT calculations (PDF)
