# Supplementary material for: Bi2CoO2F4—A Polar, Ferrimagnetic Aurivillius Oxide-Fluoride
Source: Chem Mater. 2022 Oct 17;34(21):9775–85. doi: 10.1021/acs.chemmater.2c02745 (PMC9648175; doi:10.1021/acs.chemmater.2c02745)
Supplement: Supplementary file 1 — cm2c02745_si_001.pdf [file cm2c02745_si_001.pdf]

$Bi_2CoO_2F_4$ – a polar, ferrimagnetic Aurivillius oxide-fluoride

Euan A. S. Scott,[1] Eleni Mitoudi Vagourdi,[2] Mats Johnsson,[2] Vanessa Cascos,[1] Filbin John,[1] Dave Pickup,[1] Alan V. Chadwick,[1] Hania Djani,[3] Eric Bousquet,[4] Weiguo Zhang,[5] P. Shiv Halasyamani,[5] Emma E. McCabe,*[1,6]

[1] School of Physical Sciences, University of Kent, Canterbury, Kent, CT2 7NH, U. K.

[2] Department of Materials and Environmental Chemistry, Stockholm University, SE-106 91 Stockholm, Sweden

[3] Centre de Développement des Technologies Avancées, cité 20 aout 1956, Baba Hassan, Alger, Algeria

[4] Theoretical Materials Physics, Q-MAT, CESAM, Université de Liège, Allée 6 août, 17, B-4000, Sart Tilman, Belgium

[5] Department of Chemistry, University of Houston, 112 Fleming Building, Houston TX 77204, U. S. A.

[6] Department of Physics, Durham University, South Road, Durham, DH1 3LE, U. K.

* Corresponding author: emma.mccabe@durham.ac.uk

Supplementary information:

SI1 Analysis of variable temperature XRPD data

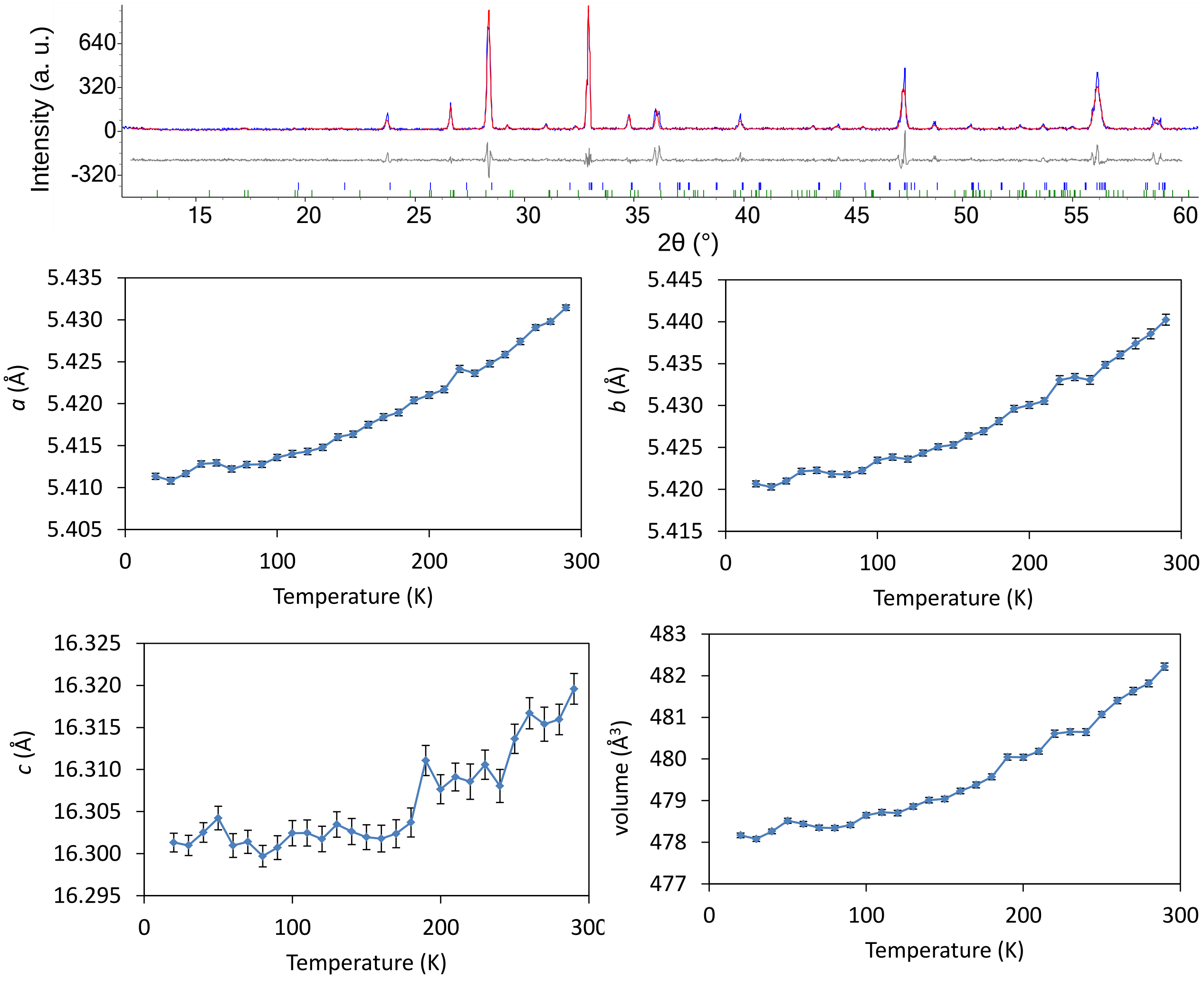

Figure 1 (a) Rietveld refinement profile for refinement using 20 K XRPD data for $Bi_2CoO_2F_4$ using *Pbca* model with upper blue and lower green ticks indicating peak positions for $Bi_2CoO_2F_4$ (86.3(9)%) and $Bi_7F_{11}O_5$ (13.7(9)% by mass), respectively; R_{wp} = 23.2%, R_p = 18.1% and χ^2 = 1.88%.

SI2 Pawley refinements to index 50 K NPD data collected for $Bi_2CoO_2F_4$

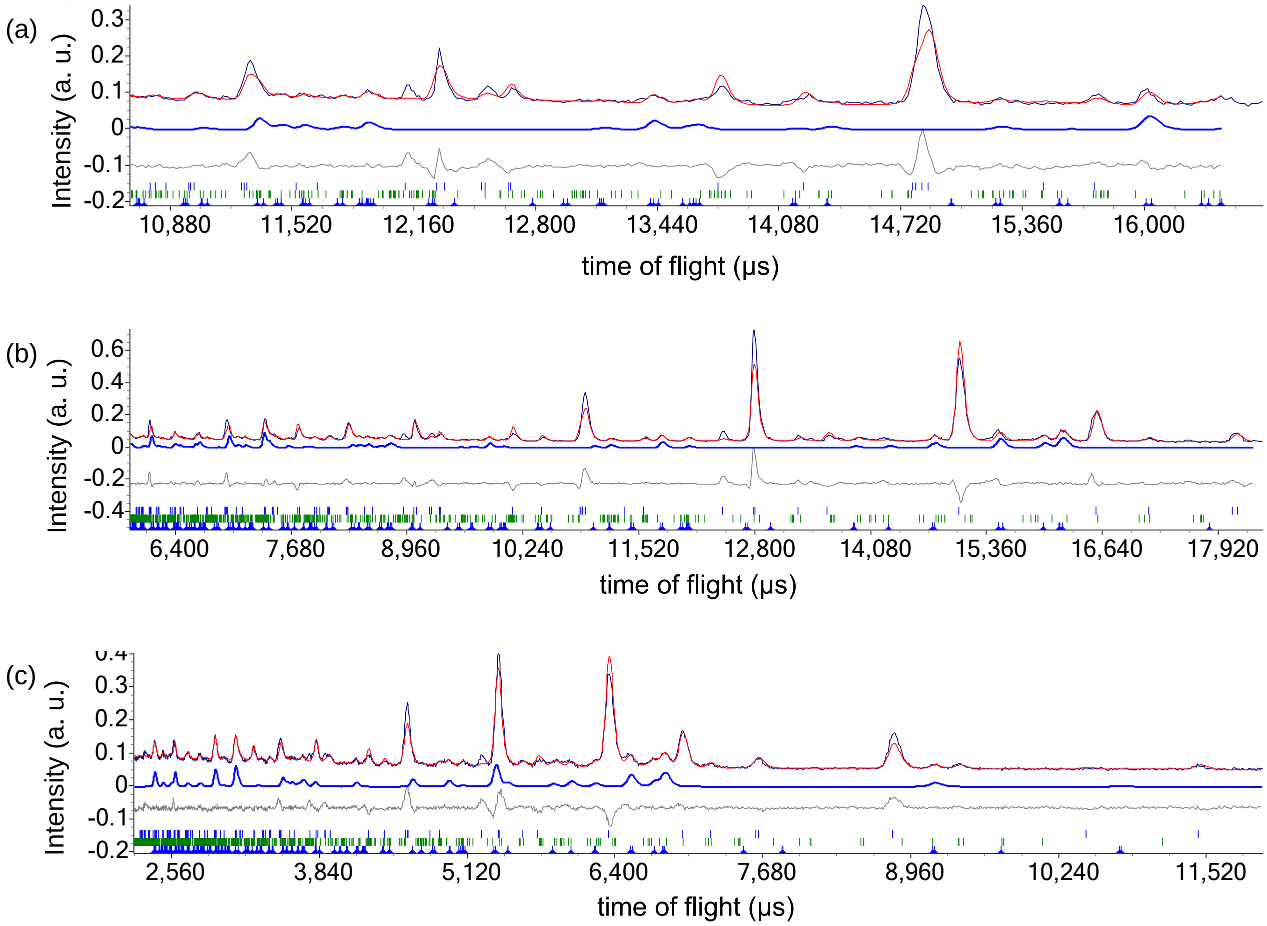

Figure 2 Refinement profiles using NPD data collected at 50 K for $Bi_2CoO_2F_4$ with the ideal aristotype *I*4/*mmm* model (with unit cell $a_t \times a_t \times c_t$) included as a Rietveld phase (upper ticks), an impurity phase $Bi_7F_{11}O_5$ (middle green ticks) and Pawley phase for a larger $\sqrt{2}a_t \times \sqrt{2}a_t \times c_t$ unit cell of *P*4 symmetry (bottom blue ticks) with intensity from this phase highlighted in blue. Panel (a) shows data from the 154° bank, panel (b) the 91° bank and (c) the 35° bank.

SI3 Symmetry analysis for *n* = 1 Aurivillius phases

Schematic illustrating symmetries resulting from key structural distortions of the ideal high symmetry *I*4/*mmm* model for an *n* = 1 Aurivillius phase. This analysis has been carried out using ISODISTORT[1] and with reference to Hatch et al.[2]

- out-of-phase rotation about $[110]_t$ ($a^-a^-c^0$) is described by irrep X_3^+ (ϕϕ0 ϕϕ0).
- out-of-phase rotation about $[110]_t$ ($a^-a^-c^0$) changing sense from block to block is described by X_4^+ (ϕϕ0 –ϕ-ϕ0), inconsistent with peaks observed in these diffraction data.
- rotation about $[001]_t$ ($a^0a^0c^\pm$) is described by X_2^+ (00θ 00θ).
- in-plane polar displacements along $[110]_t$ are described by Γ_5^-.
- in-plane antipolar displacements M_5^+.

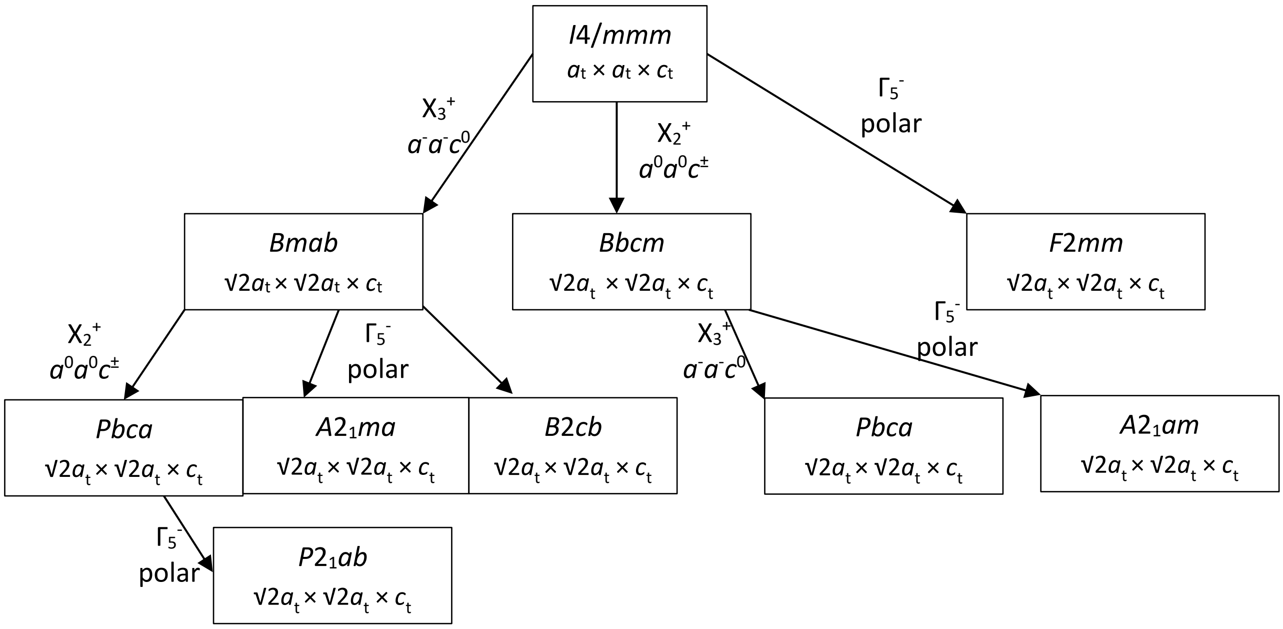

Figure 3 Symmetry map showing octahedral rotation distortions (X_2^+, X_3^+) and in-plane displacements (Γ_5^-); non-standard setting of space groups are used to give the long axis along [001].

SI4 Mode inclusion analysis for $Bi_2CoO_2F_4$ using 50 K NPD data starting from ideal high symmetry *I4/mmm* model

The symmetry-adapted distortion mode approach allows a distorted structure to be described in terms of a parent, high symmetry structure acted on my symmetry adapted distortion modes, the amplitudes of which can be refined. These distortion modes can describe displacements or occupancies of sites, or magnetic moments on sites.[1] We've used a "mode inclusion" analysis method to see the change in fit between observed and calculated diffraction patterns as a result of simulated annealing when the amplitudes of individual modes, or groups of modes as allowed by symmetry, are allowed to refine.[3-5] This method can be powerful if different distorted models cannot be clearly distinguished by indexing, e.g. if reflection conditions are similar.[3] The mode description of distorted structures was generated using ISODISTORT[1] and simulated annealing and refinements were carried out using Topas Academic software.[6, 7] Starting from the high symmetry *I4/mmm* model for $Bi_2CoO_2F_4$ (allowing Γ_1^+ mode amplitudes to refine, which give no change in symmetry), groups of symmetry adapted distortion modes were included in the refinement in turn and their amplitudes refined in the simulated annealing. This mode inclusion analysis was carried out using the 91° bank of data. The R_{wp} of the best solution from the simulated annealing was output, these modes were then removed from the refinement (their amplitudes fixed at zero) and the next group of modes were introduced.

(a)

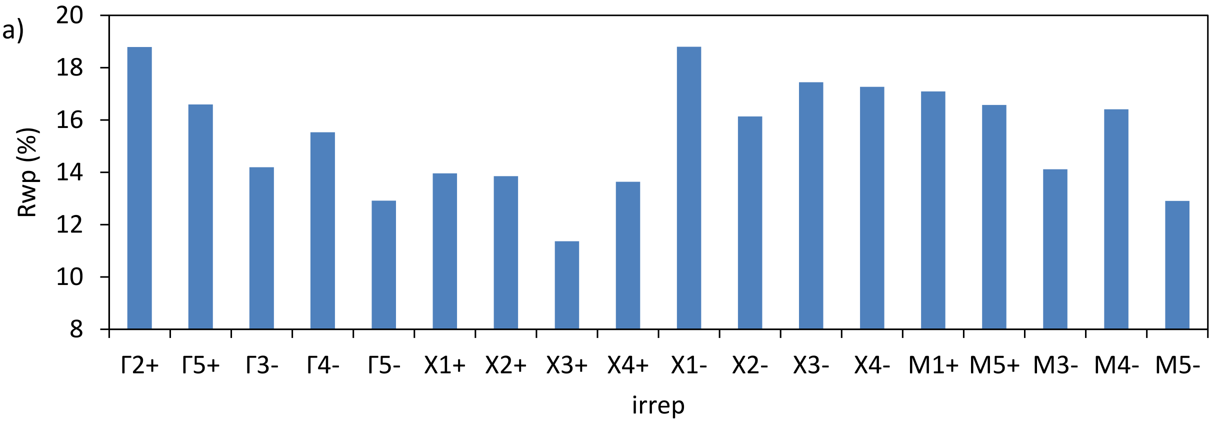

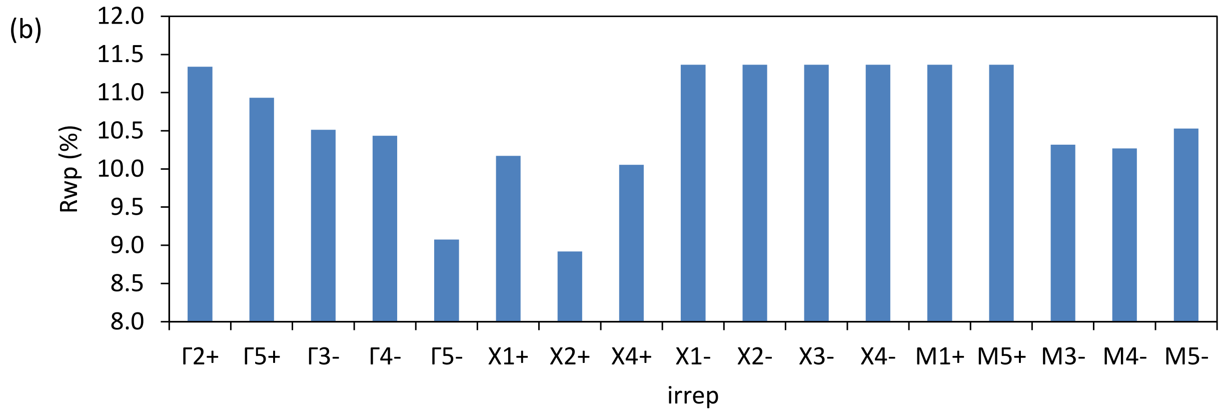

Figure 4 Details of mode inclusion analysis showing R_{wp} from simulated annealing as (a) each group of modes is individually introduced into the distortion mode refinement and (b) X3+ mode amplitudes allowed to refine as other groups of modes are introduced individually to the distortion mode refinement.

After this first round of mode inclusion analysis (Figure 4(a)), the X_3^+ modes (which lower the symmetry to *Bmab*, *a-cb* setting of space group 54, *Cmca*) were found to give the greatest improvement in fit (R_{wp} decreased from ~18.8% to 11.4%). A model of *Bmab* symmetry allows rotation of CoX_6 octahedra about $[110]_t$ ($a^-a^-c^0$ in Glazer notation, or ϕϕ0 ϕϕ0 in the notation of Hatch et al).[2] However, this model gave an unreasonably high atomic displacement parameter for the equatorial *X*(1) (0.094(2) Å^2). Atomic displacement parameters are often correlated with site occupancies in refinements and so this could indicate vacancies on this site, or a problem with the position of the site. Allowing the atomic displacement parameter to refine anisotropically suggested displacement of this *X*(1) site in the *ab* plane. Disordering this *X*(1) ion from the high symmetry 8*e* site to a general 16*g* position improves the fit (decreasing R_{wp} from 6.97% to 6.56% for two additional parameters) and may indicate some disorder (either static or dynamic) of *X*(1) within the *ab* plane. However, even in this disordered model, the atomic displacement parameter for this ion is still twice that of the next highest site (see Table 1 of the Supporting Information).

A second round of mode inclusion analysis, with X_3^+ mode amplitudes free to refine, indicated that the fit improved significantly if X_2^+ or Γ_5^- mode amplitudes were refined (Figure 4(b)), with R_{wp} decreasing to 8.9% or to 9.1% for X_2+ or Γ_5- modes, respectively.

X_2^+ modes allow rotation of CoX_6 octahedra about $[001]_t$ and lower the symmetry further to *Pbca*, allowing X_3^+ and X_2^+ rotations (i.e. $a^-a^-c^\pm$, or ϕϕθ ϕϕθ in the notation of Hatch et al).[2] This second octahedral rotation is consistent with the high atomic displacement parameter for *X*(1) refined for the ordered *Bmab* model. This *Pbca* model gives a good fit, but only a very slight improvement compared with the disordered *Bmab* model (R_{wp} decreased to 6.51% for four additional parameters) and in the absence of clear reflections forbidden by *Pbca* symmetry, it is hard to confirm this further symmetry lowering and additional ordered rotation mode from our powder diffraction data.

Γ_5^- modes describe polar displacements in along $[110]_t$ and, combined with X_3^+ rotations, could give models of *B*2*cb* (*cab* setting of space group 41, *Aba*2) or of *A*2$_1$*ma* symmetry. These models differ as to whether the X_3^+ rotations are about polar axis (*B*2*cb*), or about an axis perpendicular to the polar axis (*A*2$_1$*ma*). Given the negligible orthorhombic distortion, these two models are almost indistinguishable and we show here the *B*2*cb* model, analogous to that reported for Bi_2WO_6 between 660 – 960°C.[8]

Given the high atomic displacement parameter found for *X*(1) in both ordered and disordered *Bmab* models, and the polar *B*2*cb* model, its important to also consider polar models allowing rotation of CoX_6 octahedra about both in-plane (X_3^+ rotations) and about $[001]_t$ (X_2^+) axes. Taking the non-polar *Pbca* model allowing both X_3^+ and X_2^+ rotations, and allowing in-plane polar displacements (Γ_5^- distortions) lowers the symmetry further to *P*2$_1$*ab* symmetry (*cab* setting of space group 29, *Pca*2$_1$).

SI5 Rietveld refinement for $Bi_2CoO_2F_4$ using 50 K NPD data fitted with disordered model of *Bmab* symmetry

As described in the text, disordering the O(1) ion off the high symmetry 8*e* site (¼ ¼ *z*) to a general 16*g* site (*x y z*) improves the fit noticeably (R_{wp} decreases from 6.97% (55 parameters) for the ordered model, to 6.56% (57 parameters) for the disordered model). However, the equatorial O(1) site still has a noticeably high atomic displacement parameter.

Table 1 Details from Rietveld refinement using 50 K NPD data collected for $Bi_2CoO_2F_4$ using disordered *Bmab* model. The refinement was carried out using NPD data from the 91° (panel a) and 35° (panel 2) banks and included $Bi_7F_{11}O_5$ impurity (17(1)% by mass). The $Bi_2CoO_2F_4$ main phase (83(1)% by mass) had unit cell parameters *a* = 5.4344(5) Å, *b* = 5.4350(5) Å, *c* = 16.350(1) Å and volume = 482.91(7) Å^3.

**Atom**	**site**	***x***	***y***	***z***	**occupancy**	**U_{iso} × 100 (Å^3)**
Bi	8*f*	0	0.0074(5)	0.3274(1)	1	0.54(4)
Co	4*a*	0	0	0	1	0.3(1)
O/F(1)	16*g*	0.298(2)	0.297(2)	0.5313(3)	0.5	3.4(1)
O/F(2)	8*i*	0	-0.0755(7)	0.1228(2)	1	1.7(1)
O/F(3)	8*e*	0.25	0.25	0.7469(3)	1	0.64(4)

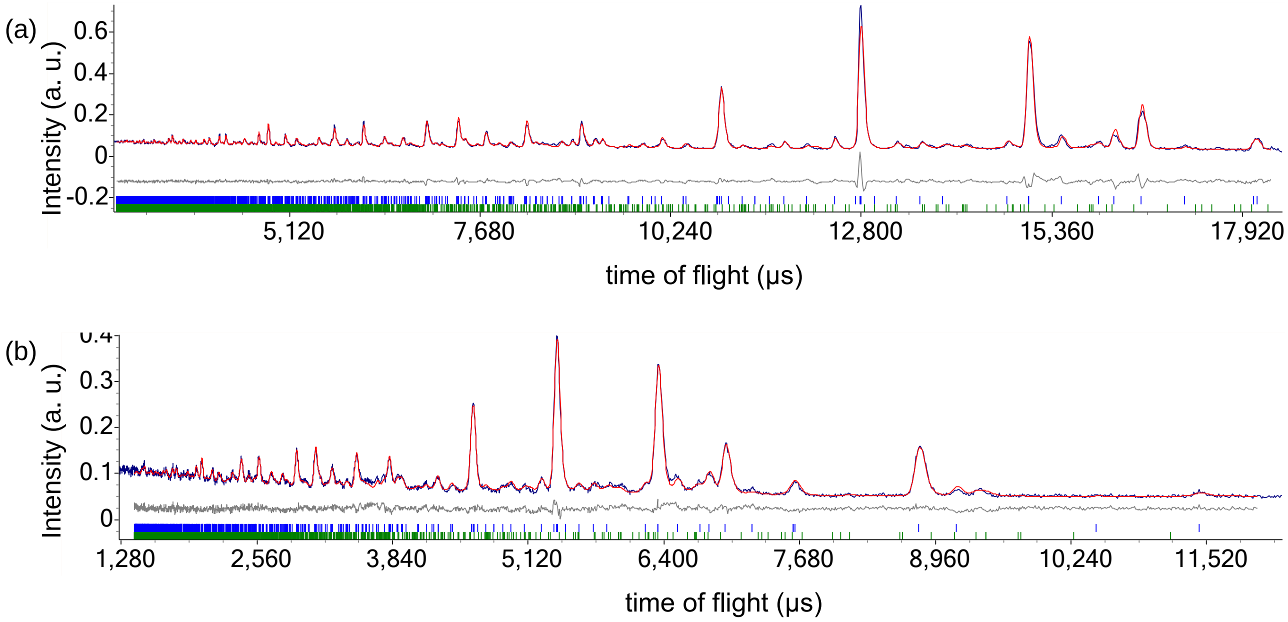

Figure 5 Rietveld refinement profiles using 50 K NPD data collected for $Bi_2CoO_2F_4$ using disordered *Bmab* model. The refinement was carried out using NPD data from the 91° (panel a) and 35° (panel 2) banks with upper ticks (blue) and lower ticks (green) showing peak positions for $Bi_2CoO_2F_4$ (83(1)% by mass) and for $Bi_7F_{11}O_5$ (17(1)% by mass), respectively; R_{wp} = 6.56%, R_p = 4.72% and χ^2 = 3.11%.

SI6 Rietveld refinement for $Bi_2CoO_2F_4$ using 50 K NPD data fitted with model of *Pbca* symmetry

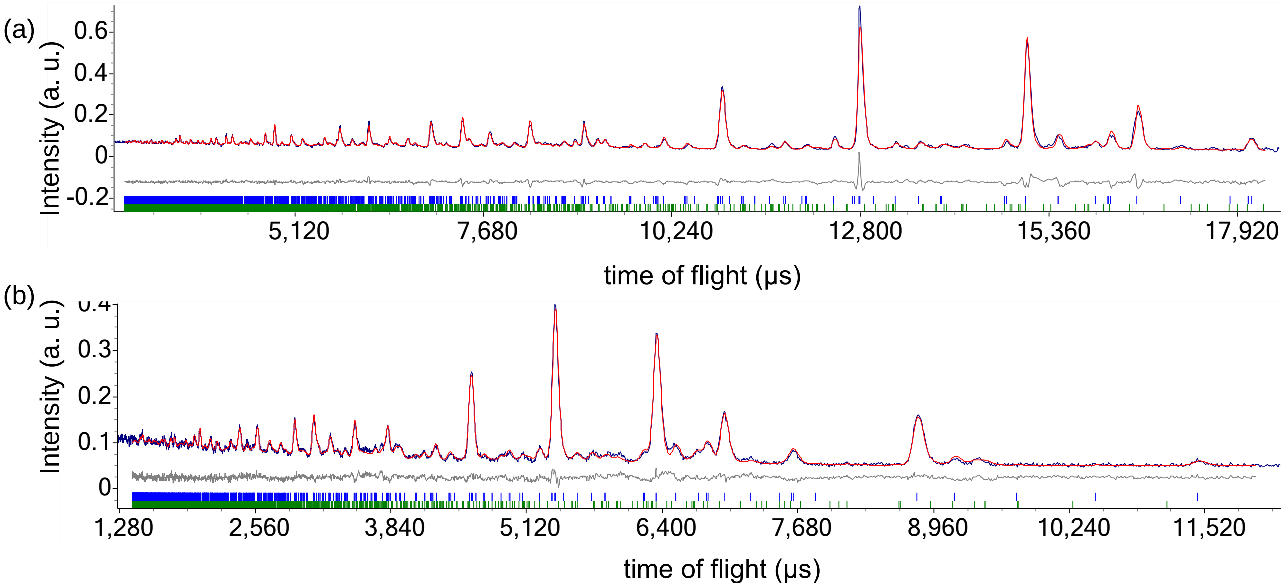

Figure 6 Rietveld refinement profiles using 50 K NPD data collected for $Bi_2CoO_2F_4$ using *Pbca* model. The refinement was carried out using NPD data from the 91° (panel a) and 35° (panel b) banks with upper ticks (blue) and lower ticks (green) showing peak positions for $Bi_2CoO_2F_4$ (83(1)% by mass) and for $Bi_7F_{11}O_5$ (17(1)% by mass), respectively; R_{wp} = 6.50%, R_p = 4.69% and χ^2 = 3.08%.

Table 2 Details from Rietveld refinement using 50 K NPD data collected for $Bi_2CoO_2F_4$ using *Pbca* model. The refinement was carried out using NPD data from the 91° and 35° banks and included $Bi_7F_{11}O_5$ impurity (17(1)% by mass). The $Bi_2CoO_2F_4$ main phase (83(1)% by mass) had unit cell parameters *a* = 5.4344(6) Å, *b* = 5.4342(6) Å, *c* = 16.350(1) Å and volume = 482.85(8) Å^3.

**Atom**	**site**	***x***	***y***	***z***	**occupancy**	**U_{iso} × 100 (Å^3)**
Bi	8*c*	-0.0091(6)	-0.001(1)	0.3271(1)	1	0.50(4)
Co	4*a*	0	0	0	1	0.1(1)
O/F(1)	8*c*	0.792(1)	0.299(1)	0.0293(3)	1	4.4(1)
O/F(2)	8*c*	0.0700(9)	-0.004(2)	0.1228(2)	1	2.5(1)
O/F(3)	8*c*	0.741(1)	0.248(1)	0.2471(3)	1	0.64(5)

SI7 Rietveld refinement for $Bi_2CoO_2F_4$ using 50 K NPD data fitted with model of *B2cb* symmetry

Table 3 Details from Rietveld refinement using 50 K NPD data collected for $Bi_2CoO_2F_4$ using *B2cb* model with full site occupancies. The refinement was carried out using NPD data from the 91° and 35° banks and included $Bi_7F_{11}O_5$ impurity (14(1)% by mass). The $Bi_2CoO_2F_4$ main phase (86(1)% by mass) had unit cell parameters *a* = 5.4342(5) Å, *b* = 5.4352(5) Å, *c* = 16.348(1) Å and volume = 482.86(7) Å^3; R_{wp} = 6.64%, R_p = 4.79% and χ^2 = 3.19%.

**Atom**	**site**	***x***	***y***	***z***	**occupancy**	**U_{iso} × 100 (Å^3)**
Bi	8*b*	0.019(5)	-0.0100(5)	0.3275(1)	1	0.46(4)
Co	4*a*	0*	0	0	1	0.0(2)
O/F(1)	8*b*	0.218(5)	0.785(2)	0.5306(3)	1	5.4(2)
O/F(2)	8*b*	0.015(6)	0.0752(8)	0.1225(2)	1	1.6(1)
O/F(3)	8*b*	0.264(5)	0.756(2)	0.7466(3)	1	0.59(6)

* coordinate fixed to define the origin of the polar axis

A refinement was carried out using using a model of *B2cb* symmetry with a single global atomic displacement parameter and the occupancies of cobalt and anion sites allowed to refine. All site occupancies refined to

within an esd of unity except for O/F(1) site. This site occupancy was fixed and atomic displacement parameters for individual sites were then refined (Figure 7 and Table 4).

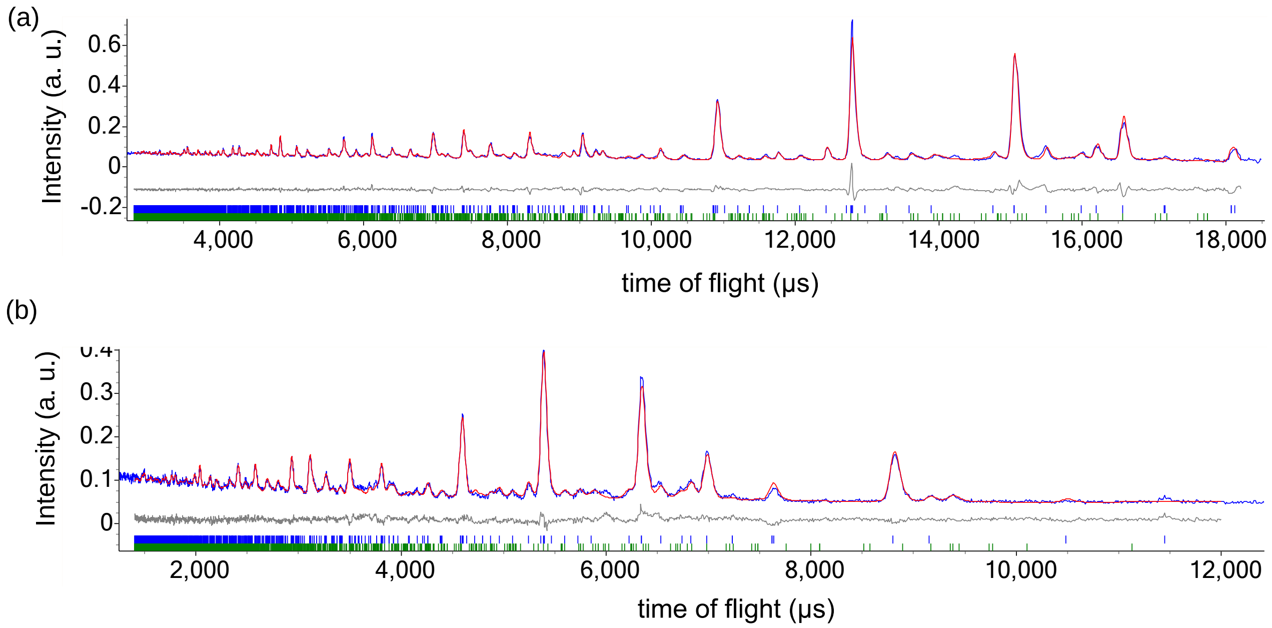

Figure 7 Rietveld refinement profiles using 50 K NPD data collected for $Bi_2CoO_2F_4$ using *B*2*cb* model with anion vacancies. The refinement was carried out using NPD data from the 91° (panel a) and 35° (panel b) banks with upper ticks (blue) and lower ticks (green) showing peak positions for $Bi_2CoO_2F_4$ (85(1)% by mass) and for $Bi_7F_{11}O_5$ (15(1)% by mass), respectively; R_{wp} = 6.64%, R_p = 4.84% and χ^2 = 3.18%.

Table 4 Details from Rietveld refinement using 50 K NPD data collected for $Bi_2CoO_2F_4$ using *B*2*cb* model with anion vacancies. The refinement was carried out using NPD data from the 91° and 35° banks and included $Bi_7F_{11}O_5$ impurity (15(1)% by mass). The $Bi_2CoO_2F_4$ main phase (85(1)% by mass) had unit cell parameters *a* = 5.4345(5) Å, *b* = 5.4349(5) Å, *c* = 16.349(1) Å and volume = 482.90(7) Å^3; R_{wp} = 6.64%, R_p = 4.84% and χ^2 = 3.18%.

**Atom**	**site**	***x***	***y***	***z***	**occupancy**	**U_{iso} × 100 (Å^3)**
Bi	8*b*	0.012(6)	-0.0105(5)	0.3279(1)	1	0.46(4)
Co	4*a*	0*	0	0	1	0.2(2)
O/F(1)	8*b*	0.213(6)	0.787(1)	0.5319(4)	0.68	1.0(1)
O/F(2)	8*b*	0.007(7)	0.0770(7)	0.1231(2)	1	1.6(1)
O/F(3)	8*b*	0.258(6)	0.753(1)	0.7465(3)	1	0.52(5)

* coordinate fixed to define the origin of the polar axis

SI8 Rietveld refinement for $Bi_2CoO_2F_4$ using 50 K NPD data fitted with model of *P*2$_1$*ab* symmetry

Table 5 Details from Rietveld refinement using 50 K NPD data collected for $Bi_2CoO_2F_4$ using *P*2$_1$*ab* model with fully occupied anion sites. The refinement was carried out using NPD data from the 91° and 35° banks and included $Bi_7F_{11}O_5$ impurity (14(1)% by mass). The $Bi_2CoO_2F_4$ main phase (86(1)% by mass) had unit cell parameters a = 5.4343(5) Å, b = 5.4343(6) Å, c = 16.350(1) Å and volume = 482.84(8) Å^3 R_{wp} = 6.28%, R_p = 4.57% and χ^2 = 2.87%.

Atom	site	*x*	*y*	*z*	occupancy	U_{iso} × 100 (Å^3)
Bi(1)	4*a*	0.014(5)	0.010(1)	0.0768(4)	1	1.2(1)
Bi(2)	4*a*	0.513(5)	0.007(2)	0.5772(4)	1	1.9(1)
Co	4*a*	0*	-0.019(4)	0.747(1)	1	0.5(2)
O/F(1)	4*a*	0.211(6)	0.292(2)	0.7852(5)	1	1.5(1)
O/F(2)	4*a*	0.768(9)	0.228(8)	0.258(2)	1	15.9(9)
O/F(3)	4*a*	0.019(7)	-0.081(2)	0.874(1)	1	3.6(4)
O/F(4)	4*a*	0.506(7)	-0.064(2)	0.3723(9)	1	3.3(4)
O/F(5)	4*a*	0.262(6)	0.241(3)	-0.0062(6)	1	2.0(2)
O/F(6)	4*a*	0.758(6)	0.253(3)	0.4994(6)	1	1.4(1)

* coordinate fixed to define the origin of the polar axis

A refinement was then carried out using a single global atomic displacement parameter and the occupancies of cobalt and anion sites allowed to refine. All site occupancies refined to within an esd of unity except for the anion sites O/F(1), (2) and (4). These site occupancies were fixed and atomic displacement parameters for individual sites were then refined (Figure 8 and Table 9).

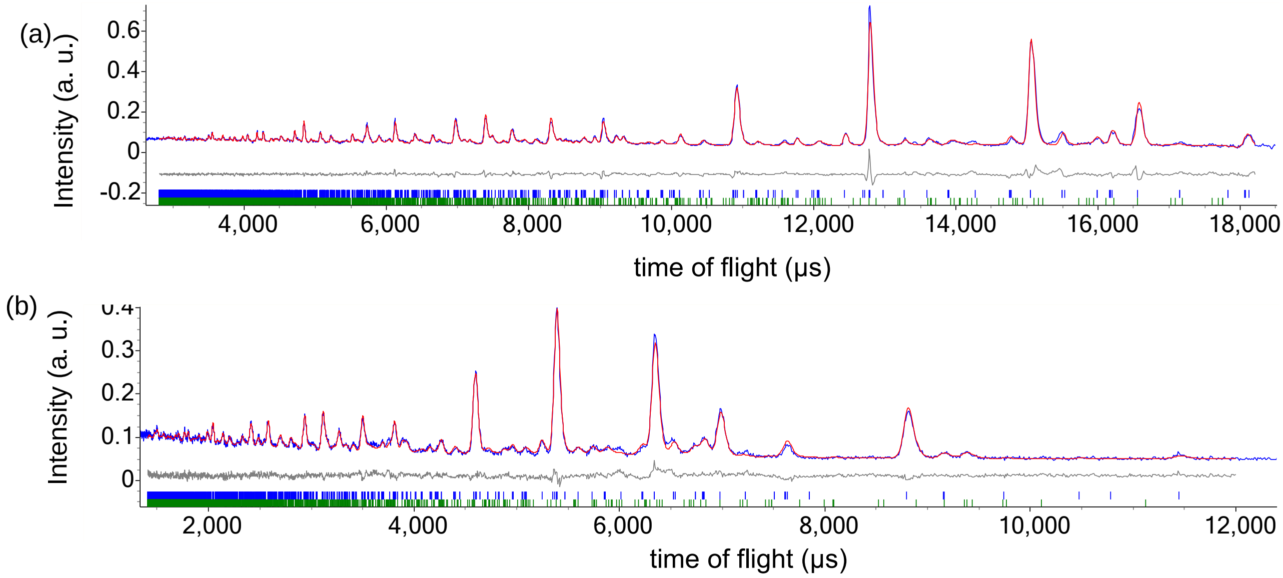

Figure 8 Rietveld refinement profiles using 50 K NPD data collected for $Bi_2CoO_2F_4$ using *P*2$_1$*ab* model with anion vacancies. The refinement was carried out using NPD data from the 91° (panel a) and 35° (panel b) banks with upper ticks (blue) and lower ticks (green) showing peak positions for $Bi_2CoO_2F_4$ (86(1)% by mass) and for $Bi_7F_{11}O_5$ (14(1)% by mass), respectively; R_{wp} = 6.32%, R_p = 4.64% and χ^2 = 2.90%.

Table 9 Details from Rietveld refinement using 50 K NPD data collected for $Bi_2CoO_2F_4$ using $P2_1ab$ model with anion vacancies. The refinement was carried out using NPD data from the 91° and 35° banks and included $Bi_7F_{11}O_5$ impurity (14(1)% by mass). The $Bi_2CoO_2F_4$ main phase (86(1)% by mass) had unit cell parameters a = 5.4343(5) Å, b = 5.4339(6) Å, c = 16.350(1) Å and volume = 482.81(8) Å^3; R_{wp} = 6.32%, R_p = 4.64% and χ^2 = 2.90%.

**Atom**	**site**	***x***	***y***	***z***	**occupancy**	**U_{iso} × 100 (Å^3)**
Bi(1)	4*a*	0.004(6)	0.011(2)	0.0776(7)	1	1.7(2)
Bi(2)	4*a*	0.505(6)	0.009(2)	0.5771(7)	1	1.4(1)
Co	4*a*	0*	-0.024(4)	0.747(1)	1	0.6(2)
O/F(1)	4*a*	0.208(7)	0.290(3)	0.7865(7)	0.75	1.5(2)
O/F(2)	4*a*	0.806(7)	0.199(4)	0.2700(9)	0.76	2.7(4)
O/F(3)	4*a*	0.027(7)	-0.071(4)	0.874(1)	1	2.3(3)
O/F(4)	4*a*	0.517(7)	-0.067(4)	0.373(1)	0.81	2.0(4)
O/F(5)	4*a*	0.255(7)	0.243(3)	-0.0060(6)	1	1.7(3)
O/F(6)	4*a*	0.749(7)	0.256(3)	0.4996(6)	1	1.4(2)

* coordinate fixed to define the origin of the polar axis

SI9 Bond lengths and bond valence sum analysis $Bi_2CoO_2F_4$ models refined from 50 K NPD data

Table 10 Selected bond lengths and angles from Rietveld refinement using 50 K NPD data for models of *B*2*cb* and $P2_1ab$ symmetries.

B2cb bond lengths (in Å) and angles		$P2_1ab$ bond lengths (in Å) and angles	
Bi – O(1)	1 ×2.81(2)	Bi(1) – O(1)	1 ×2.76(2)
Bi – O(2)	1 ×2.380(5)	Bi(1) – O(3)	1 ×2.41(2)
Bi – O(2)	1 ×2.83(5)	Bi(1) – O(3)	1 ×2.73(5)
Bi – O(2)	1 ×2.88(5)	Bi(1) – O(3)	1 ×2.97(5)
Bi – O(3)	1 ×2.29(3)	Bi(1) – O(5)	1 ×2.27(4)
Bi – O(3)	1 ×2.30(3)	Bi(1) – O(5)	1 ×2.30(4)
Bi – O(3)	1 ×2.31(3)	Bi(1) – O(5)	1 ×2.31(4)
Bi – O(3)	1 ×2.33(3)	Bi(1) – O(5)	1 ×2.34(4)
Co – O(1)	2 ×2.00(3)	Bi(2) – O(2)	1 ×2.94(3)
Co – O(1)	2 ×2.02(2)	Bi(2) – O(4)	1 ×2.44(3)
Co – O(2)	2 ×2.057(4)	Bi(2) – O(4)	1 ×2.80(5)
Co – O(1) – Co	145.8(7)°	Bi(2) – O(4)	1 ×2.91(5)
		Bi(2) – O(6)	1 ×2.26(4)
		Bi(2) – O(6)	1 ×2.28(4)
		Bi(2) – O(6)	1 ×2.28(4)
		Bi(2) – O(6)	1 ×2.37(4)
		Co – O(1)	1 ×2.13(4)
		Co – O(1)	1 ×2.14(4)
		Co – O(2)	1 ×1.87(4)
		Co – O(2)	1 ×1.94(4)
		Co – O(3)	1 ×2.09(3)
		Co – O(4)	1 ×2.09(4)
		Co – O(1) – Co	140.7(1)°
		Co – O(2) – Co	149.5(2)°

Bond valence sum calculations for the anion sites in the *B*2*cb* model gave apparent valences of ~0.9-1.0 for equatorial (1) and apical (2) sites, and values of 1.7-2.2 for fluorite site O(3). Similarly, anion bond lengths for the $P2_1ab$ model gave apparent valences of 0.7-1.2 for equatorial sites (1) and O(2), 0.9-1.0 for apical sites O(3) and O(4) and higher values of 1.7-2.3 for the fluorite O(5) and O(6) sites.

SI10 Investigation of magnetic structure from 5 K NPD data

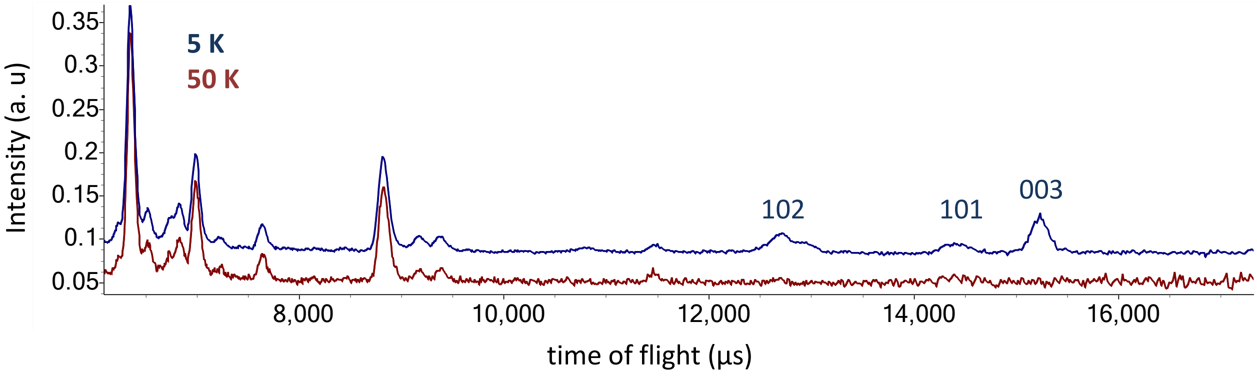

Figure 9 Extra Bragg peaks due to magnetic order observed in 5 K NPD data at long d spacing

Magnetic Bragg reflections could mostly be indexed in the same size unit cell as the nuclear structure. However, the shoulder to the 102 peak (~12700 μs, 4.52 Å, Figure 9) was not indexed by this unit cell and attempts to index this peak using larger or lower symmetry unit cells were unsuccessful. The difficulty in fitting high resolution NPD data that is thought to arise from an incommensurate modulation of the structure (see main text) may explain the reason for this magnetic peak not being fitted, and the magnetic structure analysis was carried out assuming a $P2_1ab$ nuclear structure. ISODISTORT was used to derive descriptions of magnetic structures in terms of symmetry-adapted magnetic ordering modes and mode inclusion analysis[3-5] was used to consider possible magnetic structures.

The most significant improvements in fit came from collinear antiferromagnetic structures with moments oriented either along [100] (the polar *a* axis and the axis about which CF_6 octahedra rotate) – referred to as the mΓ_1 magnetic structure ($P2_1ab$, 29.99); or along [010], referred to as the mΓ_2 magnetic structure ($P2_1a'b'$, 29.103) (Figure 10). Given the tiny orthorhombic distortion, these two models give very similar fits and we can't distinguish between them from these NPD data. Some anisotropic broadening of magnetic Bragg reflections was observed and is thought to arise from antiphase boundaries[5, 9] in the magnetic structure perpendicular to the *c* axis with a magnetic correlation length $\xi_c \approx 60(10)$ Å at 5 K.

Whilst the collinear magnetic structures illustrated in Figure 10 give good fits to the data, the symmetry lowering also allows non-collinear magnetic structures: for the mΓ_1 model, further AFM components along [010] and [001] are also allowed, whilst for the mΓ_2 model, an AFM component along [001] (out-of-plane) and an in-plane [100] ferromagnetic component are allowed.

For the mΓ_1 model, allowing the second in-plane AFM component to refine did not improve the fit and this parameter was too strongly correlated with the primary in-plane component along [100] to give a stable refinement. Allowing the out-of-plane AFM component to refine gave negligible improvement in fit but a small out-of-plane component, giving a total ordered moment of 2.4(1) μ_B per Co^{2+} site (2.3(1) μ_B along [100] and 0.7(2) μ_B along [001]). Refinement details are shown in Table 11 and the refinement profiles and nuclear and magnetic structure are shown in Figures 11 and 12.

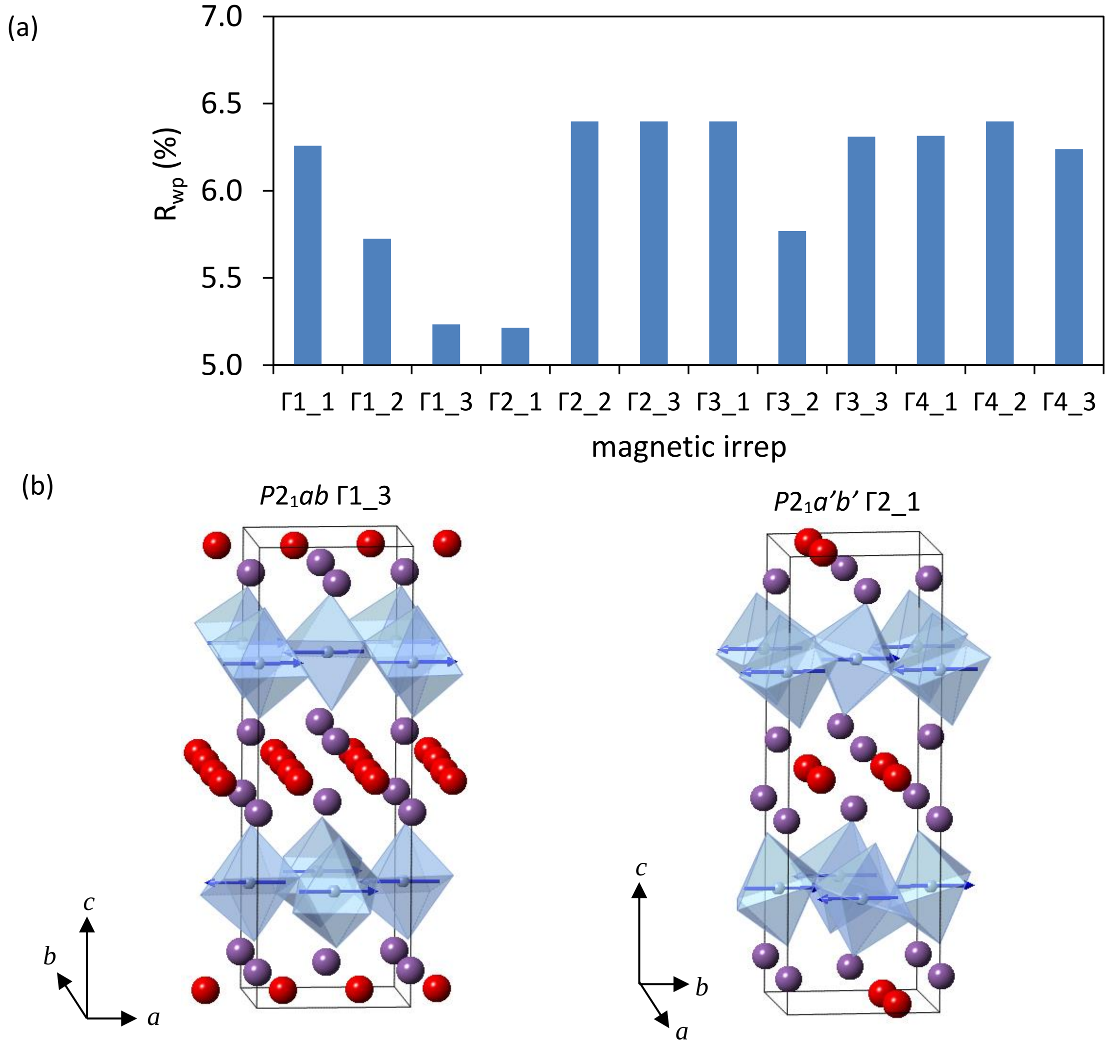

Figure 10 (a) shows results from mode inclusion analysis to determine magnetic structure, assuming $P2_1ab$ nuclear structure, and (b) gives schematic representations of collinear mΓ_1 and mΓ_2 magnetic structures (F sites omitted for clarity).

Table 11 Details from Rietveld refinement using 5 K NPD data collected for $Bi_2CoO_2F_4$ using $P2_1ab$ model (with anion vacancies) and mΓ1 magnetic model. The refinement was carried out using NPD data from the 91° and 35° banks and included $Bi_7F_{11}O_5$ impurity (14% by mass). The $Bi_2CoO_2F_4$ main phase (86% by mass) has unit cell parameters a = 5.4317(5) Å, b = 5.4314(5) Å, c = 16.3485(9) Å and volume = 482.31(7) Å^3 with Co^{2+} moments of 2.4(1) μ_B (2.3(1) μ_B along a and 0.7(2) μ_B along c); R_{wp} = 5.85%, R_p = 3.98% and χ^2 = 14.3%.

**Atom**	**site**	***x***	***y***	***z***	**occupancy**	**U_{iso} × 100 (Å^3)**
Bi(1)	4*a*	0.006(5)	0.010(2)	0.0779(6)	1	0.3(1)
Bi(2)	4*a*	0.508(5)	0.010(2)	0.5769(6)	1	0.3(1)
Co	4*a*	0*	-0.013(4)	0.750(2)	1	0.0(1)
F(1)	4*a*	0.208(6)	0.290(3)	0.7850(7)	0.75	0.1(1)
F(2)	4*a*	0.806(6)	0.198(4)	0.2711(8)	0.76	1.6(3)
F(3)	4*a*	0.025(5)	-0.070(4)	0.873(1)	1	1.3(3)
F(4)	4*a*	0.519(6)	-0.069(5)	0.374(1)	0.81	0.9(4)
O(5)	4*a*	0.252(5)	0.241(2)	-0.0060(5)	1	0.5(2)
O(6)	4*a*	0.743(5)	0.254(2)	0.4991(6)	1	0.1(1)

* coordinate fixed to define the origin of the polar axis

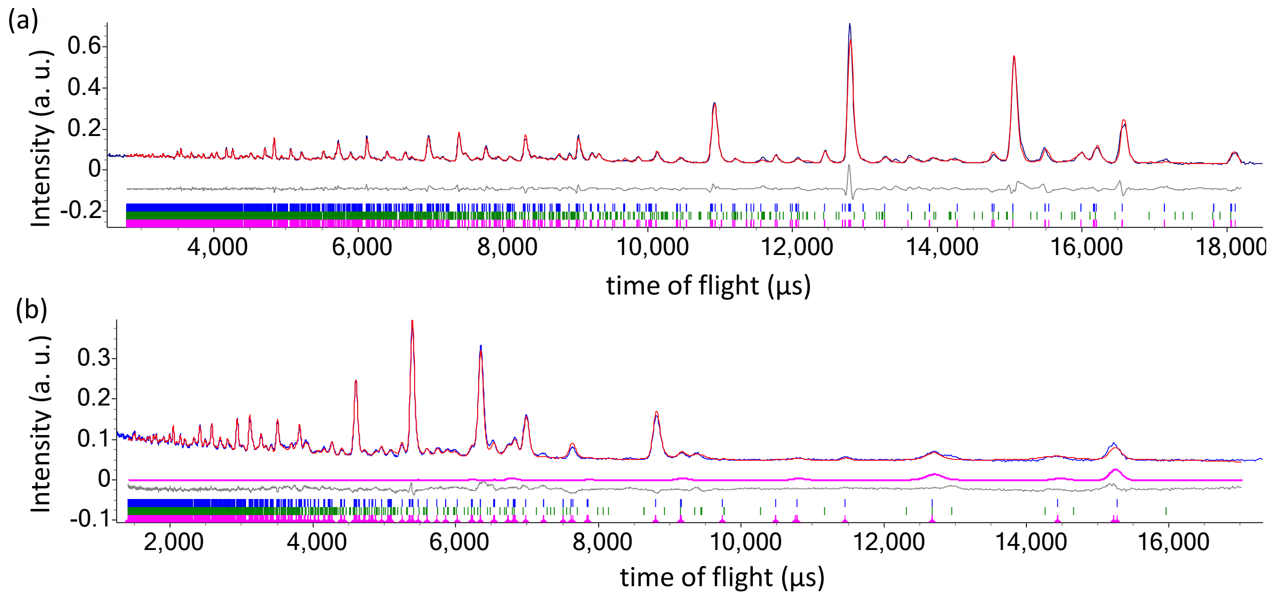

Figure 11 Rietveld refinement profiles using 5 K NPD data collected for $Bi_2CoO_2F_4$ using $P2_1ab$ model (with anion vacancies) and mΓ1 magnetic model. The refinement was carried out using NPD data from the 91° (panel a) and 35° (panel b) banks with upper ticks (blue) and middle ticks (green) showing peak positions for $Bi_2CoO_2F_4$ (86% by mass) and for $Bi_7F_{11}O_5$ (14% by mass), respectively; bottom ticks (pink) show positions of magnetic peaks and the magnetic scattering is highlighted in pink. R_{wp} = 5.85%, R_p = 3.98% and χ^2 = 14.3%.

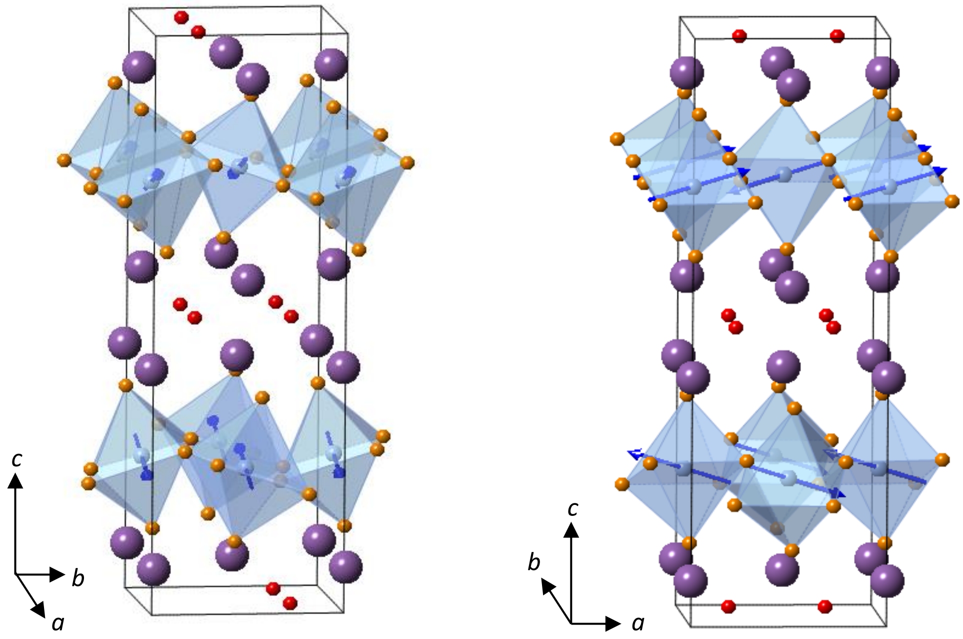

Figure 12 Schematic illustration of $P2_1ab$ nuclear structure and $m\Gamma_1$ magnetic structure from refinement using 5 K NPD data. CoF_6 polyhedra are shown in pale blue and Co^{2+} moments are shown by blue arrows; Co, Bi, F and O sites are shown by blue, purple, orange and red spheres, respectively.

For the $m\Gamma_2$ model, allowing the out-of-plane AFM component to refine did not improve the fit and this parameter refined to a value close to zero with a large esd. Allowing the out-of-plane AFM component to refine gave negligible improvement in fit but refining the in-plane FM component did improve the fit, giving a total ordered moment of 2.5(1) μ_B per Co^{2+} site (2.37(7) μ_B along [010] and 0.7(2) μ_B along [100]).

Refinement details are shown in Table 12 and the refinement profiles and nuclear and magnetic structure are shown in Figures 13 and 14.

Table 12 Details from Rietveld refinement using 5 K NPD data collected for $Bi_2CoO_2F_4$ using $P2_1ab$ model (with anion vacancies) and mΓ2 magnetic model. The refinement was carried out using NPD data from the 91° and 35° banks and included $Bi_7F_{11}O_5$ impurity (14% by mass). The $Bi_2CoO_2F_4$ main phase (86% by mass) has unit cell parameters a = 5.4317(5) Å, b = 5.4313(5) Å, c = 16.348 (1) Å and volume = 482.30(7) Å^3 with Co^{2+} moments of 2.5(1) μ_B (2.37(1) μ_B along b and 0.7(2) μ_B along a); R_{wp} = 5.83%, R_p = 3.94% and χ^2 = 14.2%.

**Atom**	**site**	***x***	***y***	***z***	**occupancy**	**U_{iso} × 100 (Å^3)**
Bi(1)	4*a*	0.006(4)	0.010(2)	0.0780(5)	1	0.1(1)
Bi(2)	4*a*	0.508(5)	0.009(2)	0.5767(6)	1	0.5(1)
Co	4*a*	0*	-0.020(4)	0.748(1)	1	0.2(2)
F(1)	4*a*	0.207(5)	0.290(3)	0.7847(7)	0.75	0.0(1)
F(2)	4*a*	0.805(6)	0.198(4)	0.2712(9)	0.76	1.9(4)
F(3)	4*a*	0.024(5)	-0.072(3)	0.873(1)	1	1.3(3)
F(4)	4*a*	0.519(6)	-0.067(4)	0.374(1)	0.81	0.9(4)
O(5)	4*a*	0.252(5)	0.241(2)	-0.0059(6)	1	0.5(2)
O(6)	4*a*	0.743(5)	0.254(2)	0.4991(6)	1	0.1(1)

* coordinate fixed to define the origin of the polar axis

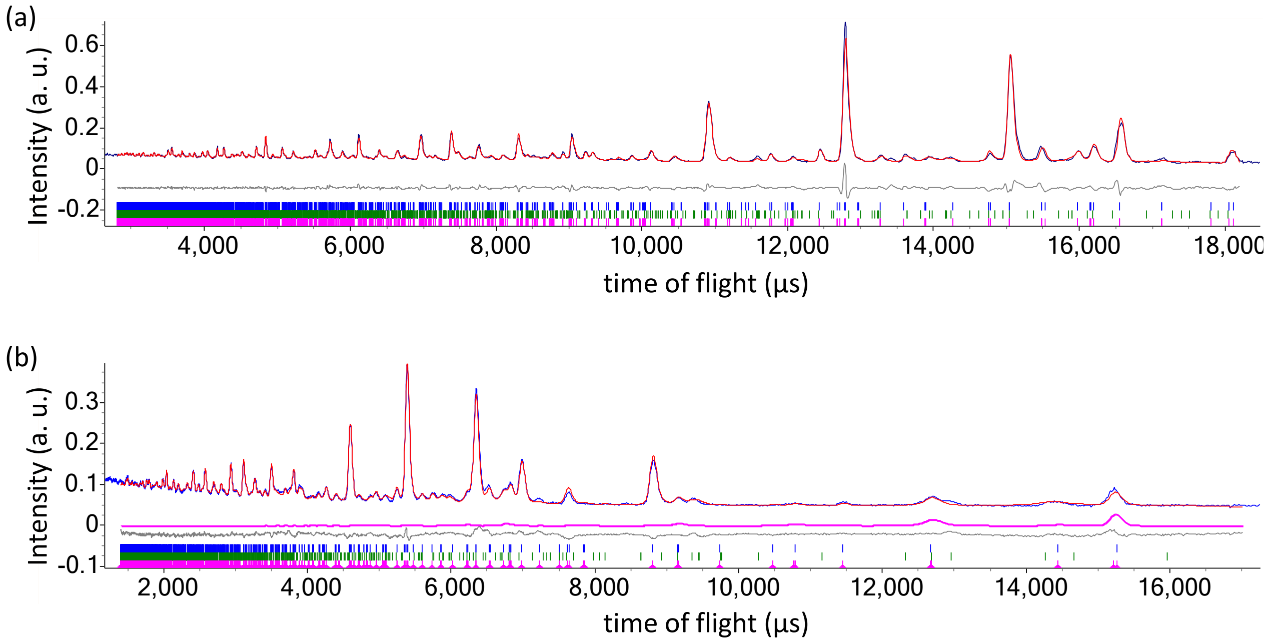

Figure 13 Rietveld refinement profiles using 5 K NPD data collected for $Bi_2CoO_2F_4$ using $P2_1ab$ model (with anion vacancies) and mΓ2 magnetic model. The refinement was carried out using NPD data from the 91° (panel a) and 35° (panel b) banks with upper ticks (blue) and middle ticks (green) showing peak positions for $Bi_2CoO_2F_4$ (86% by mass) and for $Bi_7F_{11}O_5$ (14% by mass), respectively; bottom ticks (pink) show positions of magnetic peaks and the magnetic scattering is highlighted in pink. R_{wp} = 5.83%, R_p = 3.94% and χ^2 = 14.2%.

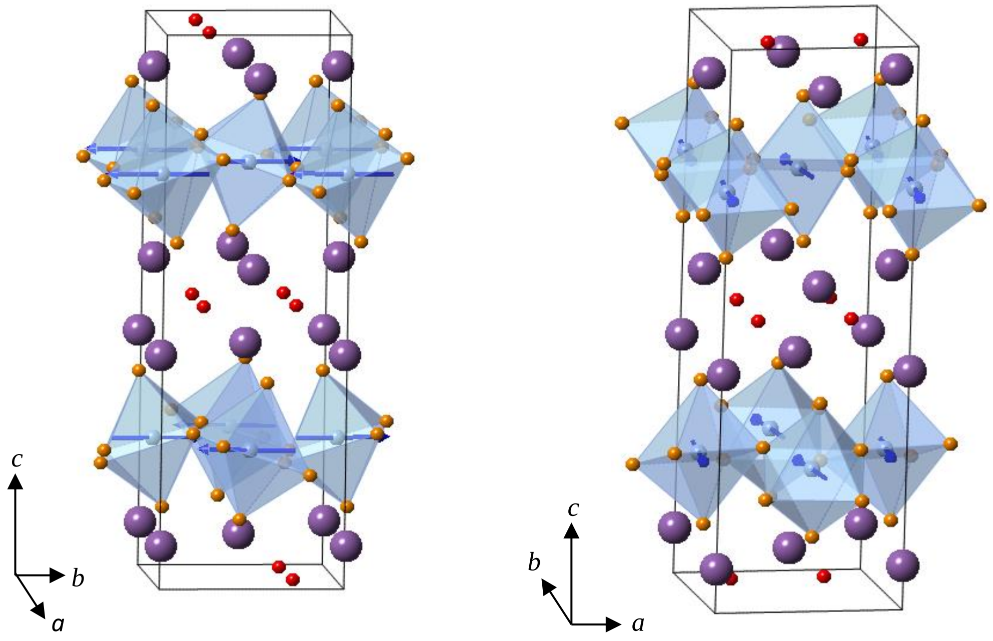

Figure 14 Schematic illustration of $P2_1ab$ nuclear structure and mΓ_2 magnetic structure from refinement using 5 K NPD data. CoF_6 polyhedra are shown in pale blue and Co^{2+} moments are shown by blue arrows; Co, Bi, F and O sites are shown by blue, purple, orange and red spheres, respectively.

A similar analysis can be carried out assuming the *B*2*cb* nuclear structure and again, the magnetic peaks are fitted fairly well by collinear magnetic structures (Figures 15 and 16).

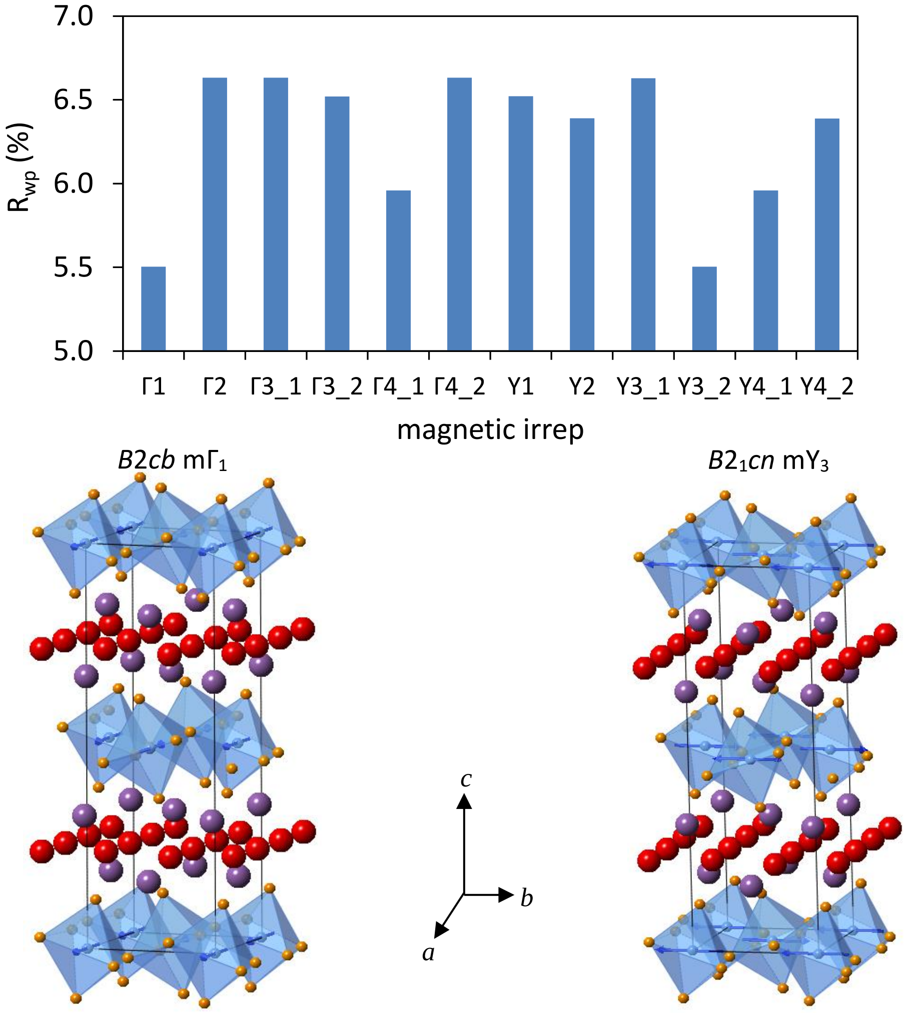

Figure 15 Results from mode inclusion analysis to determine magnetic structure, assuming *B2cb* nuclear structure, and below, schematic representations of collinear mΓ1 and mY3_2 magnetic structures.

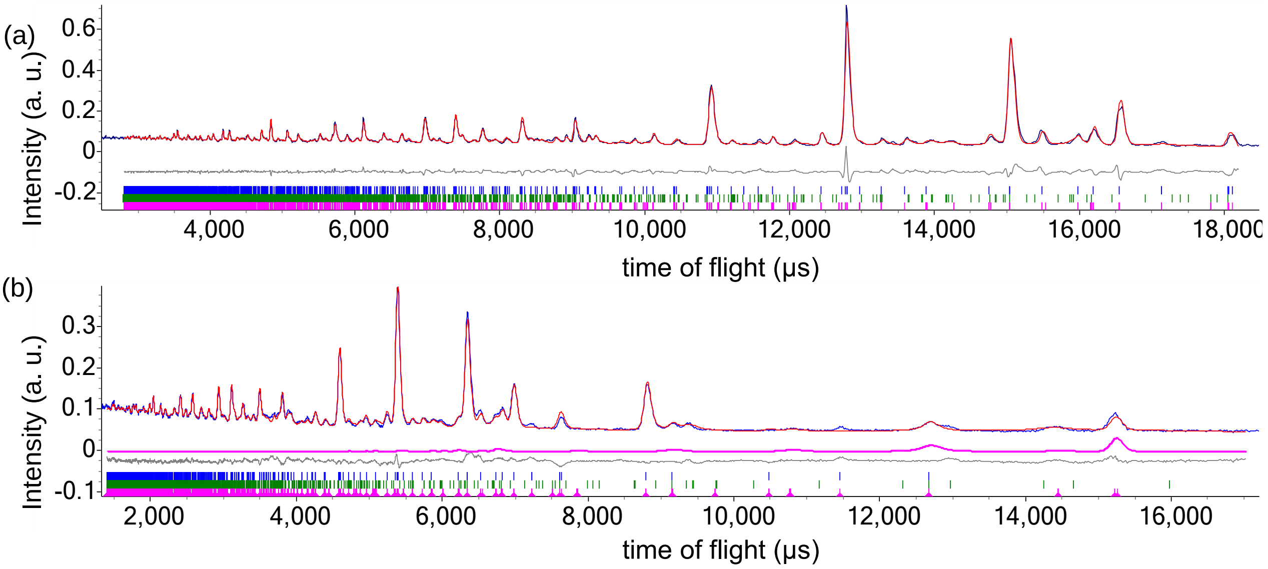

Figure 16 Rietveld refinement profiles using 50 K NPD data collected for $Bi_2CoO_2F_4$ using *B2cb* model (with anion vacancies) and mΓ1 magnetic model. The refinement was carried out using NPD data from the 91° (panel a) and 35° (panel b) banks with upper ticks (blue) and middle ticks (green) showing peak positions for $Bi_2CoO_2F_4$ (85% by mass) and for $Bi_7F_{11}O_5$ (15% by mass), respectively; ; bottom ticks (pink) show positions of magnetic peaks and the magnetic scattering is highlighted in pink. R_{wp} = 6.20%, R_p = 4.18% and χ^2 = 16.0%.

Table 13 Details from Rietveld refinement using 50 K NPD data collected for $Bi_2CoO_2F_4$ using *B*2*cb* model (with anion vacancies) and mΓ1 magnetic model. The refinement was carried out using NPD data from the 91° and 35° banks and included $Bi_7F_{11}O_5$ impurity (15% by mass). The $Bi_2CoO_2F_4$ main phase (85% by mass) had unit cell parameters *a* = 5.4317(4) Å, *b* = 5.4322(4) Å, *c* = 16.347(1) Å and volume = 482.34(6) Å^3 with Co^{2+} moments of 2.65(8) μ_B along *a*; R_{wp} = 6.20%, R_p = 4.18% and χ^2 = 16.0%.

**Atom**	**site**	***x***	***y***	***z***	**occupancy**	**U_{iso} × 100 (Å^3)**
Bi	8*b*	0.016(3)	-0.0098(5)	0.3279(1)	1	0.32(3)
Co	4*a*	0*	0	0	1	0.1(1)
O/F(1)	8*b*	0.218(3)	0.785(1)	0.5316(3)	0.68	0.6(1)
O/F(2)	8*b*	0.022(4)	0.0770(7)	0.1229(2)	1	1.6(1)
O/F(3)	8*b*	0.264(3)	0.750(1)	0.7463(3)	1	0.39(4)

* coordinate fixed to define the origin of the polar axis

In addition to the collinear AFM models shown in Figure 15, the mY3 model allows an AFM out-of-plane component but this refines to give a very small out-of-plane component with a large uncertainty in this value, and so the collinear model illustrated in Figure 15 seems more reasonable.

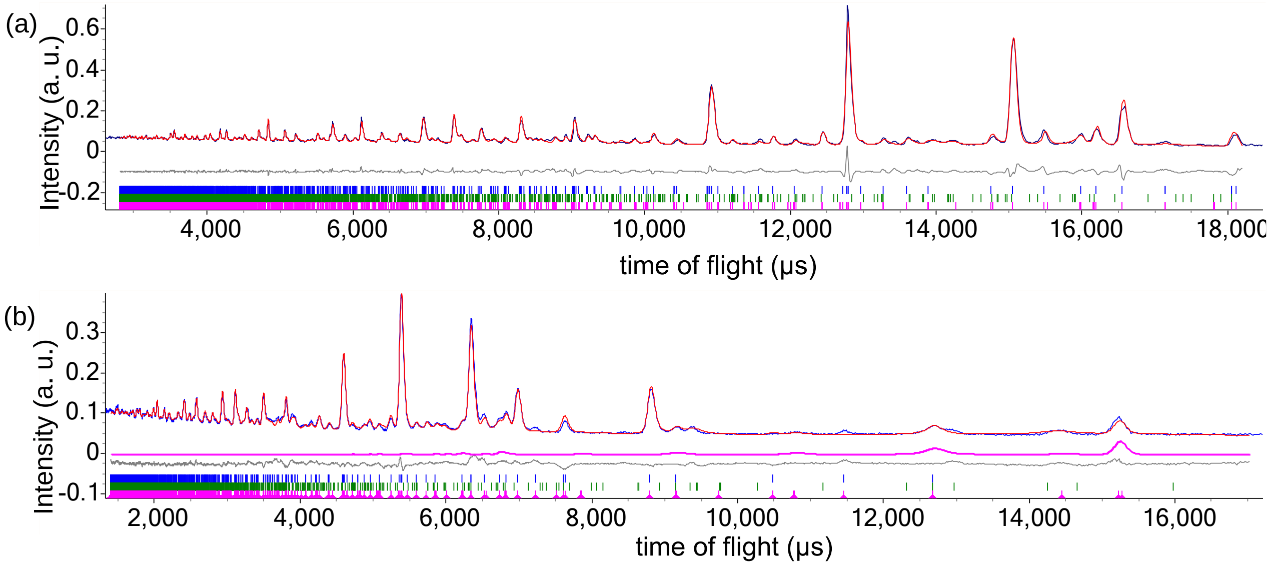

Figure 17 Rietveld refinement profiles using 50 K NPD data collected for $Bi_2CoO_2F_4$ using *B*2*cb* model (with anion vacancies) and mY_3 magnetic model. The refinement was carried out using NPD data from the 91° (panel a) and 35° (panel b) banks with upper ticks (blue) and middle ticks (green) showing peak positions for $Bi_2CoO_2F_4$ (85% by mass) and for $Bi_7F_{11}O_5$ (15% by mass), respectively; bottom ticks (pink) show positions of magnetic peaks and the magnetic scattering is highlighted in pink. R_{wp} = 6.20%, R_p = 4.18% and χ^2 = 16.0%.

Table 14 Details from Rietveld refinement using 50 K NPD data collected for $Bi_2CoO_2F_4$ using *B*2*cb* model (with anion vacancies) and mY_3 magnetic model. The refinement was carried out using NPD data from the 91° and 35° banks and included $Bi_7F_{11}O_5$ impurity (15% by mass). The $Bi_2CoO_2F_4$ main phase (85% by mass) had unit cell parameters *a* = 5.4317(4) Å, *b* = 5.4321(4) Å, *c* = 16.347(1) Å and volume = 482.33(6) Å^3 with Co^{2+} moments of 2.64(8) μ_B along *b*; R_{wp} = 6.20%, R_p = 4.18% and χ^2 = 16.0%.

**Atom**	**site**	***x***	***y***	***z***	**occupancy**	**U_{iso} × 100 (Å^3)**
Bi	8*b*	0.018(4)	-0.0098(5)	0.3278(1)	1	0.33(3)
Co	4*a*	0*	0	0	1	0.0(2)
O/F(1)	8*b*	0.220(4)	0.786(1)	0.5316(3)	0.68	0.6(1)
O/F(2)	8*b*	0.025(5)	0.0770(7)	0.1229(2)	1	1.6(1)
O/F(3)	8*b*	0.266(4)	0.750(1)	0.7463(3)	1	0.39(4)

* coordinate fixed to define the origin of the polar axis

For the *B*2*cb* nuclear structure, in addition to the mΓ_1 and mY_3 magnetic structures described above which give the best fits to the NPD data, similar magnetic structures (which give worse fits to the data) include the mY1 model (equivalent to the mΓ_1 model but with spins in the *z* = 0.5 layer reversed), and the mΓ_3 model (similar to the mY_3 but with spins in the *z*=0.5 layer reversed). Given the layered crystal structure and shorter magnetic correlation length along [001] suggesting weaker interlayer magnetic exchange interactions, these magnetic structures are likely to be similar in energy. We note that the mΓ_3 model also allows an out-of-plane component which is ferromagnetic.

SI11 Bond lengths for $Bi_2CoO_2F_4$ models refined from 5 K NPD data

Table 15 Selected bond lengths and angles from Rietveld refinement using 50 K NPD data for models of *B*2*cb* and *P*2_1*ab* symmetries.

*B*2*cb* bond lengths (in Å) and angles		*P*2_1*ab* mΓ2 bond lengths (in Å) and angles	
Bi – O(1)	1 ×2.82(1)	Bi(1) – O(1)	1 ×2.77(2)
Bi – O(2)	1 ×2.385(5)	Bi(1) – O(3)	1 ×2.41(2)
Bi – O(2)	1 ×2.83(3)	Bi(1) – O(3)	1 ×2.76(4)
Bi – O(2)	1 ×2.89(3)	Bi(1) – O(3)	1 ×2.94(4)
Bi – O(3)	1 ×2.30(1)	Bi(1) – O(5)	1 ×2.27(3)
Bi – O(3)	1 ×2.30(1)	Bi(1) – O(5)	1 ×2.29(3)
Bi – O(3)	1 ×2.31(1)	Bi(1) – O(5)	1 ×2.31(3)
Bi – O(3)	1 ×2.31(3)	Bi(1) – O(5)	1 ×2.37(3)
Co – O(1)	2 ×1.99(1)	Bi(2) – O(2)	1 ×2.94(2)
Co – O(1)	2 ×2.02(1)	Bi(2) – O(4)	1 ×2.44(3)
Co – O(2)	2 ×2.055(4)	Bi(2) – O(4)	1 ×2.79(4)
Co – O(1) – Co	146.5(4)°	Bi(2) – O(4)	1 ×2.91(4)
		Bi(2) – O(6)	1 ×2.24(3)
		Bi(2) – O(6)	1 ×2.25(3)
		Bi(2) – O(6)	1 ×2.31(3)
		Bi(2) – O(6)	1 ×2.37(3)
		Co – O(1)	1 ×2.11(3)
		Co – O(1)	1 ×2.12(3)
		Co – O(2)	1 ×1.89(3)
		Co – O(2)	1 ×1.94(3)
		Co – O(3)	1 ×2.07(3)
		Co – O(4)	1 ×2.05(4)
		Co – O(1) – Co	142.5(1)°
		Co – O(2) – Co	149.0(2)°

SI11 Density functional theory calculations

Initial phonon calculations on the parent *I*4/*mmm* model were used to determine key structural instabilities (to identify structural models for further spin-polarised calculations). Six unstable modes (which are all doubly-degenerate) were identified (Table 16).

Table 16 Summary of unstable modes found from phonon calculations for the aristotype *I*4/*mmm* model. Do these calculations give you the relative energies of the distortions? Bi, Co and X sites are shown in purple, blue and yellow, respectively.

**Mode**	**Resulting space group symmetry***	**Order parameter direction**	**Frequency (cm^{-1})**	**description**	
X_2^+	*Cmca*	(a;0)	136i	$a^0a^0c^{\pm}$ rotation of octahedra about the stacking axis	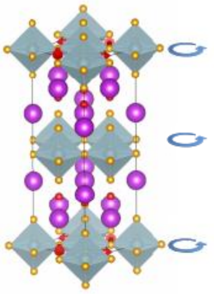
X_3^+	*Cmca*	(a;0)	98i	$a^-a^-c^0$ rotation of octahedra	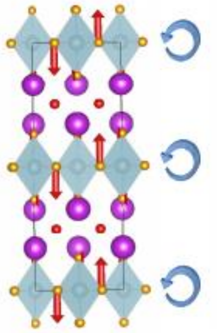
X_4^+	*Cccm*	(a;0)	84i	$a^-a^-c^0$/-($a^-a^-c^0$) rotation of octahedra	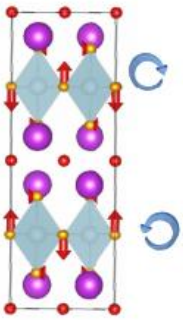
Γ_5^-	*Fmm*2	(a;a)	29i	in-plane polar displacements along [110]$_t$	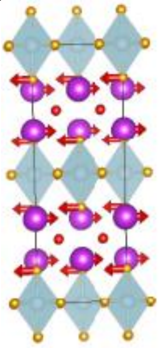

X_3^-	*Cmcm*	(a;0)	22i	in-plane antipolar displacements along $[110]_t$	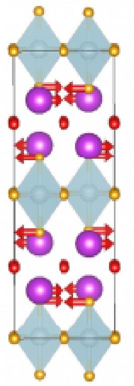
M_5^+	*Cmca*	(a;0)	13i	in-plane antipolar displacements along $[110]_t$	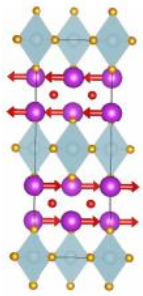

* space groups given in their standard settings

Having determined the irreps to describe the phonon instabilities of the high symmetry *I*4/*mmm* model, lower-symmetry models were taken forwards for spin-polarised calculations:

- *Pbca* (X_3+ ⊕ X_2+ ⊕ M_5+)
- *B*2*cb* (X_3+ ⊕ Γ_5-)
- *P*2$_1$*ab* (X_3+ ⊕ X_2+ ⊕ Γ_5-)

Three spin arrangements were considered for each of the three nuclear structures (see main text). Initial calculations using LDA-JTH gave the AFM2 model as the ground state for both *I*4/*mmm* and *Pbca* models (with 3 μ_B per Co^{2+} site) for *U* = 4 eV, 6 eV, and *J* = 0 eV, 0.2 eV, 0.4 eV and 0.6 eV). However, geometry optimisation of the polar models of *B*2*cb* and *P*2$_1$*ab* symmetry gave FM ground states, in contrast with experimental results. Calculations using GGA-JTH gave the FM ground state for the *I*4/*mmm* nuclear structure, but AFM2 ground states (with AFM1 next lowest in energy) for all the orthorhombic nuclear structures (for *U* = 4 eV, 6 eV; *J* = 0.2, 0.4, 0.6 eV). The energies of the three magnetic models were also compared for the three nuclear structures and the *I*4/*mmm* aristotype nuclear structure:

Table 17 Relative energies ΔE (meV per formula unit) and mode contributions to the AFM2 ground state models for $Bi_2CoO_2F_4$ relative to the *I*4/*mmm* (AFM2) model, for *U* = 4 eV and *J* = 0.6 eV.

**AFM2**		
**Nuclear structure**	**Mode contributions**	**ΔE (meV /f.u.)**
I4/mmm	-	0
Pbca	1.86 X_3+ ⊕ 1.14 X_2+ ⊕ 0.41 M_5+	-480
B2cb	1.37 X_3+ ⊕ 0.011 Γ_5-	-871
*P*2$_1$*ab*	2.02 X_3+ ⊕ 1.2 X_2+ ⊕ 0.011 Γ_5- ⊕ 0.59 M_5+	-874
**AFM1**		
**Nuclear structure**	**Mode contributions**	**ΔE (meV /f.u.)**
I4/mmm	-	-0.68
Pbca	1.84 X_3+ ⊕ 1.15 X_2+ ⊕ 0.44 M_5+	-485
B2cb	1.36 X_3+ ⊕ 0.011 Γ_5-	-794
*P*2$_1$*ab*	1.95 X_3+ ⊕ 1.25 X_2+ ⊕ 0.011 Γ_5- ⊕ 0.53 M_5+	-790
**FM**		
**Nuclear structure**	**Mode contributions**	**ΔE (meV /f.u.)**
I4/mmm	-	-82
Pbca	1.75 X_3+ ⊕ 1.25 X_2+ ⊕ 0.41 M_5+	-502
B2cb	1.35 X_3+ ⊕ 0.011 Γ_5-	-660
*P*2$_1$*ab*	2.01 X_3+ ⊕ 1.25 X_2+ ⊕ 0.011 Γ_5- ⊕ 0.50 M_5+	-648

1. Campbell, B. J.; Stokes, H. T.; Tanner, D. E.; Hatch, D. M., ISODISTORT. *J. Appl. Cryst.* **2006,** *39*, 607-614.
2. Hatch, D. M.; Stokes, H. T.; Aleksandrov, K. S.; Misjul, S. V., A2BX4. *Phys. Rev. B* **1989,** *39*, 9282.
3. McCabe, E. E.; Free, D. G.; Mendis, B. G.; Higgins, J. S.; Evans, J. S. O., Preparation, Characterization, and Structural Phase Transitions in a New Family of Semiconducting Transition Metal Oxychalcogenides β-La2O2MSe2 (M=Mn, Fe). *Chem. Mater.* **2010,** *22*, 6171-6182.
4. Tuxworth, A. J.; McCabe, E. E.; Free, D. G.; Clark, S. J.; Evans, J. S. O., Structural Characterization and Physical Properties of the New Transition Metal Oxyselenide La2O2ZnSe2. *Inorg. Chem.* **2013,** *52*, 2078-2085.
5. McCabe, E. E.; Stock, C.; Rodrigues, E. E.; Wills, A. S.; Taylor, J. W.; Evans, J. S. O., Weak spin interactions in Mott insulating La2O2Fe2OSe2. *Phys. Rev. B* **2014,** *89*, 100402(R).
6. Coelho, A. A., Topas. *J. Appl. Cryst.* **2003,** *36*, 86.
7. Coelho, A. A. *Topas Academic: General profile and structure analysis software for powder diffraction data*, Bruker AXS: Karlsruhe, Germany, 2012.
8. McDowell, N. A.; Knight, K. S.; Lightfoot, P., Unusual high temperature structural behaviour in ferroelectric Bi2WO6. *Chem. Eur. J.* **2006,** *12*, 1493-1499.
9. Her, J.-H.; Stephens, P. W.; Gao, Y.; Soloveichik, G. L.; Rijssenbeek, J.; Andrus, M.; Zhao, J.-C., Structure of unsolvated magnesium borohydride Mg(BH4)2. *Acta Cryst* **2007,** *B63*, 561-568.
